# Robustness of Protein–Ligand Binding Affinity Prediction Models to Docked and Predicted Structures

**DOI:** 10.1021/acs.jcim.6c00592

**Published:** 2026-06-23

**Authors:** Joelle N. Eaves, Daniel R. Woldring

**Affiliations:** † Department of Chemical Engineering and Materials Science, 3078 Michigan State University, East Lansing, Michigan 48824, United States; ‡ Institute for Quantitative Health Science and Engineering, 3078 Michigan State University, East Lansing, Michigan 48824, United States

## Abstract

Structure-based deep learning models for protein–ligand binding affinity prediction (PLBAP) are commonly benchmarked using experimentally resolved co-crystal structures, but real use cases often rely on computed inputs (docked or predicted complexes). To quantify this benchmark-to-deployment mismatch, we compared the CASF-2016 performance of five reproducible PLBAP pipelines across crystal structures, GNINA docking into holo/apo/AlphaFold3-predicted receptors, and AlphaFold3 co-folding. Critically, access to an experimentally resolved apo receptor conformer provided only marginal benefit over AlphaFold3-predicted receptor structures. AlphaFold3 co-folding was competitive with, and for some models significantly better than, rigid-receptor docking into the apo conformer (Holm *p* ≤ 0.006). Multipose averaging showed source-dependent effects and did not recover near-crystal performance for any perturbed input. Interaction-level profiling revealed distinct shifts in protein–ligand interaction distributions that help explain the observed decrease in predictive performance. These findings provide practical guidance for two audiences: (1) model developers should consider reporting performance on benchmarks extending beyond crystal structures to more appropriately reflect deployment settings, and (2) end users should expect performance differences depending on structure generation method and pose selection.

## Graphical abstract

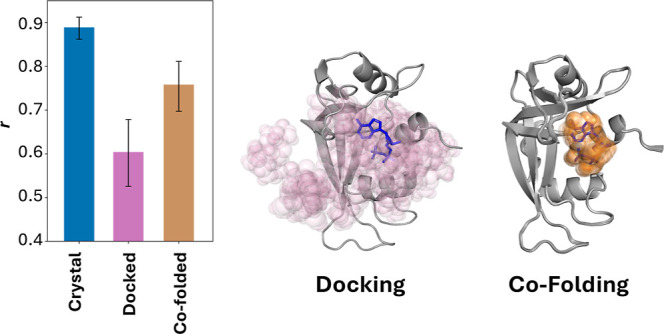

## Introduction

Deep learning models for protein–ligand binding affinity prediction (PLBAP) have become widely used in structure-based drug discovery, virtual screening, and lead optimization. The performance of novel PLBAP models is typically judged on curated benchmarking data sets such as CASF-2016, which contains experimentally resolved protein–ligand co-crystal structures.^1,2^ While crystal-based benchmarks provide a useful basis for standardized cross-model comparison, they seldom reflect the computationally generated structures that many users rely on in practice.^3^ This creates a benchmark-to-deployment mismatch that can obscure both the model robustness and practical utility.

In practical drug discovery workflows, experimentally resolved co-crystal structures for the protein–ligand pair of interest are rarely available. Instead, PLBAP models are often applied to computationally generated protein–ligand complexes. Structure generation methods include popular molecular docking programs (e.g., AutoDock Vina derivatives such as GNINA^4^) and modern structure prediction methods like AlphaFold3.^5^ These computationally generated structures can differ from crystal structures in both the global conformation and local interaction geometry. We refer to these differences from the crystal reference as structural perturbations. In practice, these perturbations are introduced by the exact workflow the user chooses (e.g., apo docking versus co-folding), which means expected PLBAP performance is workflow-dependent.

To make the comparison across structure sources more explicit, we distinguish two common deployment paradigms. First, rigid-receptor docking keeps the receptor fixed and samples ligand poses in a defined binding region. This paradigm is widely used for throughput and is frequently applied to either a holo (co-crystal-derived) receptor or an experimentally resolved apo receptor conformer when only the apo structure is available. Second, co-folding methods attempt to generate a full protein–ligand complex structure directly and often produce a ranked ensemble of candidate complexes. These paradigms should be represented in benchmarking because they define distinct input distributions seen by end users.

Evaluating PLBAP robustness to structural perturbations is essential for determining whether crystal-benchmark performance translates to deployment.^6^ This robustness is important to understand, as perturbations can alter the geometry and composition of key protein–ligand interactions that many structure-based PLBAP models depend upon. Moreover, many workflows generate multiple candidate poses per complex and either select a single best-ranked pose or aggregate predictions across pose ensembles. However, the effect of pose-ensemble aggregation on cross-model PLBAP performance has not, prior to this study, been characterized across models. Accordingly, our analyses evaluate both single-pose performance and pose-ensemble behavior across multiple structure-generation workflows.

Herein, we address a benchmark-to-deployment mismatch in protein–ligand binding affinity prediction by evaluating five leading, reproducible PLBAP pipelines across structure sources that reflect both ideal benchmark conditions and common real-world workflows. Specifically, we compare performance on (i) experimental co-crystal complexes, (ii) GNINA docking into apo crystal receptors, (iii) GNINA docking into holo crystal receptors, (iv) GNINA docking into AlphaFold3-predicted receptors, and (v) AlphaFold3 co-folding. This study was motivated by two practical needs. First, PLBAP model developers need evaluation protocols that reflect deployment conditions (i.e., how their models will actually be used) rather than relying exclusively on crystal co-complex benchmarks. Second, end users, including protein engineers, protein scientists, and drug discovery researchers, need realistic expectations for model performance when inputs are computationally generated (e.g., co-folded predicted complexes or docked ligands in apo/predicted receptors). By quantifying performance degradation relative to crystal inputs, comparing structure-generation workflows, and analyzing pose-ensemble effects, this work provides evidence for more deployment-relevant PLBAP benchmarking standards and clearer guidance for interpreting model performance in practice.

## Results

A central question in applied PLBAP is whether model performance reported on crystal benchmarks persists under realistic deployment conditions where co-crystals are unavailable. Commonly, PLBAP performance is reported in terms of Pearson correlation coefficient (PCC) on the CASF-2016 data set, composed of 285 co-crystal complexes with experimental binding affinities.^1^ We tested the hypothesis that PLBAP performance decreases when models are applied to computationally generated complexes that introduce perturbations, relative to the crystal reference. To assess this for the CASF-2016 benchmark, we generated protein–ligand complexes via four computational structure generation methods that introduce measurable structural perturbations relative to the experimental crystal structures. We then input both the computationally generated structures and crystals into five PLBAP models whose selection was based upon strict reproducibility criteria detailed in the Methods section: Dynaformer,^7^ EGNA,^8^ EHIGN-PLA,^9^ GIGN,^10^ and OnionNet-2.^6^ Results are organized below to first establish the nature and magnitude of structural perturbations introduced by each method. Then, we present our assessment of the impact on PLBAP performance, pose ensemble aggregation behavior, and protein–ligand interaction distributions.

### RMSD Distributions Vary Systematically by the Structure Generation Method

We hypothesized that different structure generation methods would introduce distinct, reproducible structural perturbations relative to experimental crystal complexes. Moreover, we presumed that these perturbations would correlate with differences in downstream PLBAP performance. Establishing this relationship is essential for interpreting performance differences across inputs. To quantify these perturbations, we computed three RMSD-based metrics relative to the crystal complex for each structure source: protein Cα RMSD, protein sidechain RMSD, and ligand heavy-atom RMSD (Figure 1). This analysis was performed on the single best pose per complex (that with the lowest docking score) to isolate per-method perturbations while avoiding pose-ensemble effects. RMSD calculation and alignment details are provided in Supporting Information Appendix S1. This analysis establishes that the evaluated workflows introduce distinct perturbation regimes rather than a single generic “non-crystal” condition.

**Figure 1:**
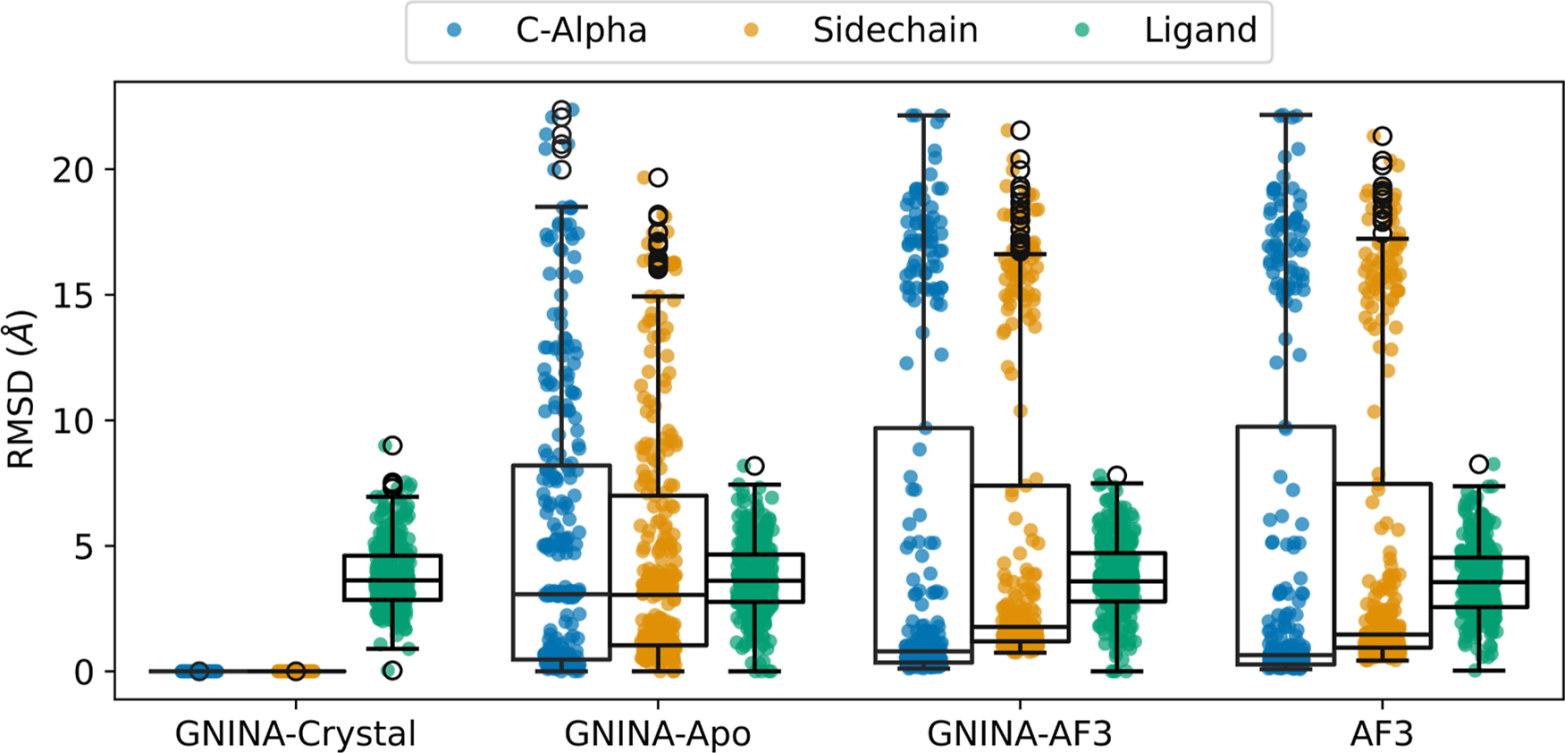
Structural perturbations relative to crystal structures across the single best poses from each structure generation method are quantified as root-mean-square deviation (RMSD). For each computational structure type, RMSD distributions are shown for protein Cα, protein sidechains, and non-hydrogen ligand atoms. GNINA-Crystal produces ligand-only perturbations, while GNINA-Apo imparted additional holo-to-apo receptor perturbations. Predicted-receptor conditions (GNINA-AF3 and AlphaFold3 co-folding) show bimodal receptor RMSD distributions, reflecting the target-dependent structural accuracy of AlphaFold3.

Because GNINA-Crystal uses the co-crystal receptor conformation, receptor RMSDs are expected to be zero by construction, isolating ligand-placement error as the dominant structural perturbation for this source. GNINA-Apo explicitly introduces receptor conformational differences (apo-to-holo), providing a controlled test of whether experimental apo conformers are sufficiently similar to holo receptors for downstream PLBAP use. The GNINA-Apo Cα RMSD distribution is approximately bell-shaped, with 42.8% of complexes below 2 Å, 35.4% between 2 and 10 Å, and 21.8% over 10 Å.

In contrast, the GNINA-AF3 and AlphaFold3 co-folding Cα RMSD distributions are strongly bimodal. The majority of complexes cluster low on the distributions, near the crystal references (67.0% and 65.3% of Cα RMSD < 2 Å, respectively). In both conditions, the upper clusters, which deviate strongly above 10 Å, are comprised of 24.9% of complexes. This bimodal structure produces medians of 0.80 Å and 0.65 Å, respectively, substantially below the GNINA-Apo median of 3.07 Å (extended statistics in Table S1). Kolmogorov–Smirnov tests confirm that both AF3-based conditions differ significantly from GNINA-Apo (both *p* < 10^–7^) but are statistically indistinguishable from each other (*p* = 0.085). Remarkably, the same 71 complexes comprise the top cluster (>10 Å Cα RMSD) in both GNINA-AF3 and AlphaFold3 co-folding. This reflects the target-dependent structural accuracy of AlphaFold3 predictions, even in co-folding.

The high similarity of these backbone RMSD distributions makes it implausible to attribute the stark performance differences (Figure 2) to receptor accuracy. Interestingly, this discrepancy is also not explained by AlphaFold3′s own structural confidence metrics: the ranking score (0.8*iPTM + 0.2*pTM) shows no significant association with per-complex prediction error.^5^ Mean interchain PAE is similarly uninformative (see Appendix S10). The narrow range of AlphaFold3 confidence scores across CASF-2016 targets is likely due to all 285 complexes falling within AlphaFold3 training data. Together, backbone RMSD and AlphaFold3 confidence metrics are insufficient to explain PLBAP performance differences at the per-complex level. Rather, as we show herein, protein–ligand interaction geometry is highly determinant of PLBAP robustness.

**Figure 2:**
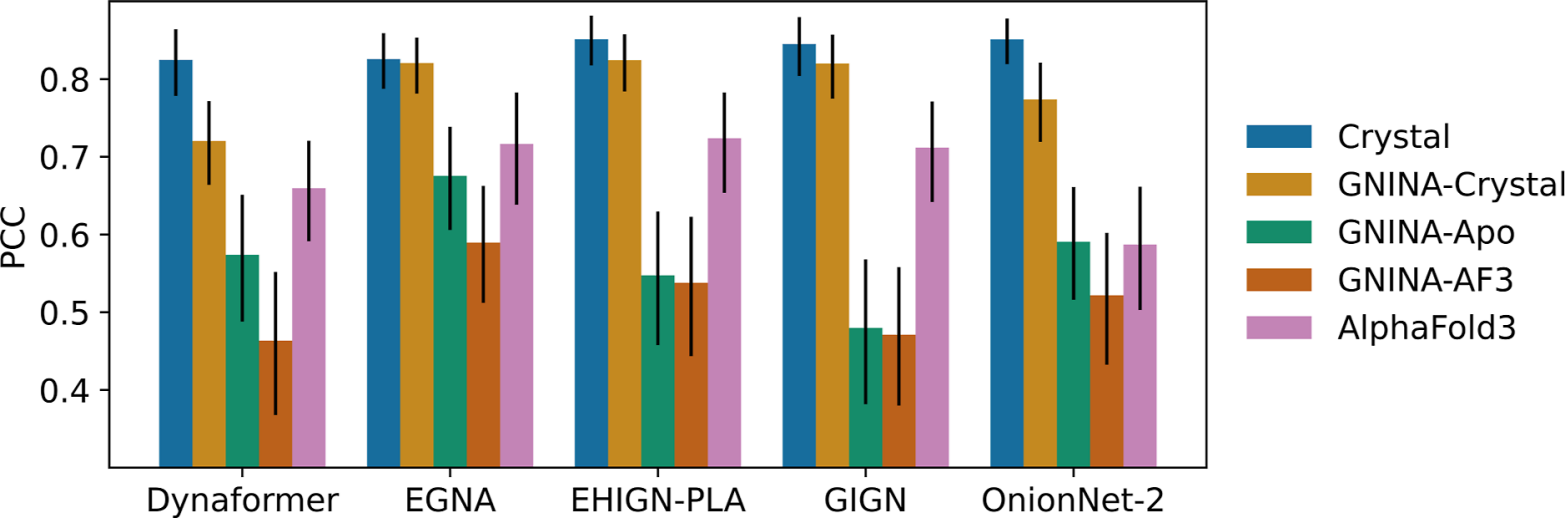
Predictive performance across investigated models for the crystal structure input versus computationally generated inputs. Crystal structures offer greatest predictive performance across all five models. Docking simulations against the crystal receptor structure by GNINA followed in performance order. Models using AlphaFold3 co-folded structures generally performed better than those using GNINA-Apo and GNINA-AF3 inputs—the results of rigid GNINA docking to Apo and AlphaFold3-generated receptors, respectively. Error bars denote 95% bootstrap confidence intervals over complexes (20,000 iterations).

### AlphaFold3 Co-folding Is Competitive with Apo-Receptor Docking for PLBAP

We next tested whether PLBAP performance depends on how the input complex is generated, with particular emphasis on a practical comparison between AlphaFold3 co-folding and rigid-receptor docking into perturbed receptor conformers (including apo structures). We evaluated five PLBAP pipelines on crystal structures and the best pose for each complex from all four computationally generated structure sources (Figure 2). We evaluated five PLBAP pipelines on crystal inputs and the single best pose from each computational source and compared PCC across structure sources using paired bootstrap resampling (*n* = 20,000) with Holm correction (Figure 2). This comparison addresses a common deployment decision: whether to use co-folding or docking when holo co-crystal structures are unavailable.

As expected, performance is highest when models are evaluated on crystal complexes (model-averaged PCC = 0.839). GNINA-Crystal, which uses the holo co-crystal receptor and therefore isolates only ligand-placement error, yielded the next-highest performance. However, GNINA-Crystal still took a performance loss relative to the crystal benchmark (model-averaged ΔPCC = 0.037, Holm *p* ≤ 0.055). This performance loss was only significant, however, for Dynaformer and OnionNet-2 (Holm *p* ≤ 0.027). This confirms that receptor conformational accuracy is a major determinant of PLBAP quality. While GNINA-Crystal provides a useful control for this study, it, however, does not provide much practical utility (i.e., if the holo crystal is available, there should be no need for redocking).

The primary question of practical importance is whether computationally generated structures can substitute for experimental ones when only apo or predicted structures are available. Direct comparison of GNINA-Apo to GNINA-Crystal reveals that docking into an experimentally resolved apo conformer substantially reduces performance relative to holo-receptor docking (model-averaged ΔPCC = 0.199, Holm *p* ≤ 0.001). This finding is notable because it demonstrates that having an experimental receptor structure does not guarantee PLBAP quality. Rather, the conformational state matters.

Crucially, AlphaFold3 co-folding outperformed GNINA-Apo for bootstrapping aggregated across models (ΔPCC = 0.105, Holm *p* ≤ 0.001). At a per-model level, too, AlphaFold3 co-folding outperformed GNINA-Apo for four of the five investigated models (significantly for EHIGN-PLA and GIGN; Holm *p* ≤ 0.021). OnionNet-2 was the sole exception. Despite the AlphaFold3 receptor being computationally generated, joint complex prediction can recover performance more effectively than rigid-receptor docking into the apo conformer. By contrast, GNINA-AF3 did not statistically differ from GNINA-Apo for both EHIGN-PLA and GIGN (ΔPCC ≤0.015, Holm p = 1.0). This suggests that the dominant source of performance loss is due to the inability of rigid-receptor docking to accommodate coupled receptor–ligand rearrangements. This result establishes AlphaFold3 co-folding as a viable, and in some cases preferred, alternative to apo-receptor docking for practical applications, such as high-throughput drug discovery.

### Averaging Predictions over Multiple Generated Poses Does Not Recover Near-Crystal Performance

Docking and co-folding typically output multiple candidate poses per complex. We hypothesized that averaging PLBAP predictions across a ranked ensemble of poses might improve performance by smoothing over individual pose errors. This has been shown to be the case in some prior works, especially for larger, more dynamic proteins, which can suffer from poor pose classification.^11,12^ However, we also considered that including lower-ranked poses could dilute the signal and that this trade-off might differ across structure generation methods. To investigate this, we computed PLBAP performance after averaging predictions across the top *N* poses, where *N* ∈ {1, 2, 3, 5, 10, 20, 30, 50, 100}, for each structure source (Figure 3).

**Figure 3:**
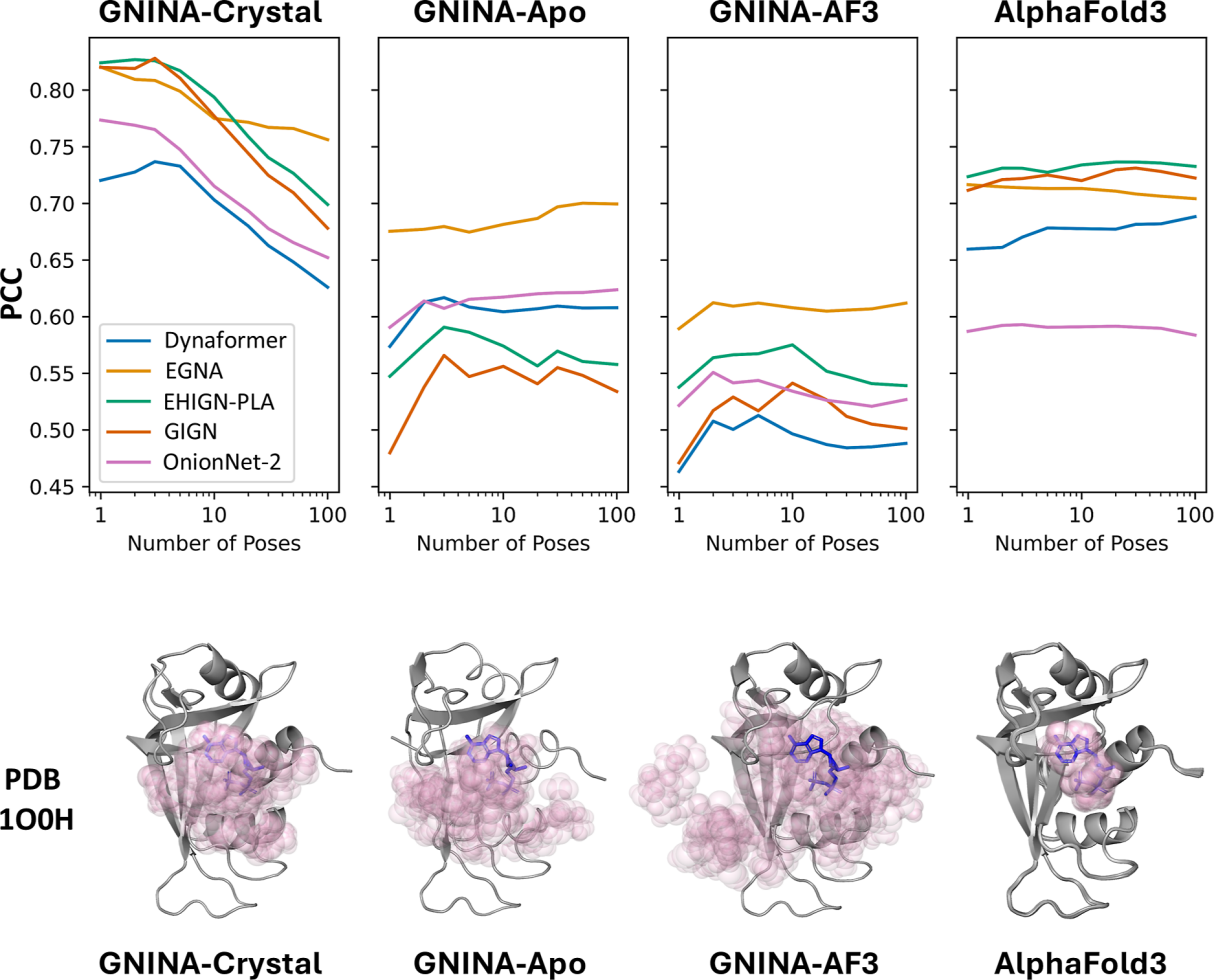
Impact of multipose prediction averaging is distinct to structure generation method. For GNINA-Crystal, performance consistently degrades with increasing N across all models. For GNINA-Apo and GNINA-AF3, performance remains low with no consistent recovery. AlphaFold3 co-folding shows minimal sensitivity to ensemble size. Below each plot are the structures of one exemplary complex from the CASF-2016 set (PDB ID 1O0H) generated by each source. The crystal ligand position is shown as a blue stick representation, while the pink lines represent the ligand positions from all 100 poses, and the protein chains are shown in gray. For each of the three GNINA docking conditions, the receptor is rigid. Conversely, for AlphaFold3 co-folding, protein chains are shown for all 100 generated poses.

Multipose prediction averaging for GNINA-Crystal consistently reduced performance of all five PLBAP models as *N* increased. A rapid, steady decrease was observed beyond *N* = 3 poses. The mean PCC drop from *N* = 1 to *N* = 100 was 0.109 ± 0.027 (standard deviation) averaged across models. Furthermore, the Spearman correlations between PCC and *N* were strongly and uniformly negative for all five models (ρ ∈ [−1.0, −0.85]; all *p* ≤ 0.005). This suggests that lower-ranked docking poses introduce ligand placements that increasingly dilute the signal of the best pose. This is clear, too, from visual inspections of the GNINA-Crystal structures (e.g., 1O0H in Figure 3), where ligand position across all 100 poses can deviate noticeably from the crystal-binding mode.

For GNINA-Apo and GNINA-AF3, performance remained low across ensemble sizes, with no consistent recovery attributable to pose averaging. A modest improvement is observed when averaging from *N* = 1 to *N* ≈ 3 poses for GNINA-Apo and GNINA-AF3, though this trend is model-dependent and does not recover near-crystal performance. For users of these pipelines, averaging over the top 3 poses may offer some benefit. Unlike GNINA-Crystal, Spearman correlations differed substantially across models (GNINA-Apo: ρ ∈ [−0.25, 0.98], GNINA-AF3: ρ ∈ [−0.23, −0.05]). This indicates that any apparent trends for these methods are model-dependent. For AlphaFold3 co-folding, ensemble averaging had negligible effects across all *N*, with the mean PCC gain from *N* = 1 to *N* = 100 being 0.001 ± 0.014 (standard deviation). This indicates that AlphaFold3 poses are broadly similar in their utility for downstream PLBAP regardless of their relative ranking. Visual assessment of PDB 1O0H generated by AlphaFold3 co-folding (Figure 3) aligns with the observation that all 100 poses are visually similar to one another. These findings caution PLBAP model users from assuming multipose averaging will be beneficial and motivate optimization for each use case.

### Protein–Ligand Interaction Distributions Shift across Structure Sources

Differences between multiple structures are often discussed in terms of RMSD, which captures global structural deviation but does not directly reflect the local interaction patterns that PLBAP models depend upon. Therefore, we profiled protein–ligand interactions across structure sources to identify mechanistic differences associated with robustness losses. We hypothesized that different structure generation methods would produce distinct shifts in protein–ligand interaction profiles and that these shifts would help explain the observed performance differences.

We profiled all poses using the Protein–Ligand Interaction Profiler (PLIP),^13^ a tool for quantifying protein–ligand interaction information (e.g., angles, distance) that has been widely adopted for predictive modeling and mechanistic investigations.^14–16^ We parsed PLIP outputs into per-pose counts for all major interaction classes: hydrogen bonds, hydrophobic contacts, pi-stacking, pi–cation interactions, halogen bonds, and salt bridges (see Appendix S9). We then compared interaction count distributions across pose sources to the crystal reference, both for the single best poses and for up to 100 total poses per complex (Figure 4). Pairwise comparisons of total interaction count distributions were performed using two-sided Mann–Whitney U tests with Benjamini–Hochberg false discovery rate correction across all source pairs.

**Figure 4:**
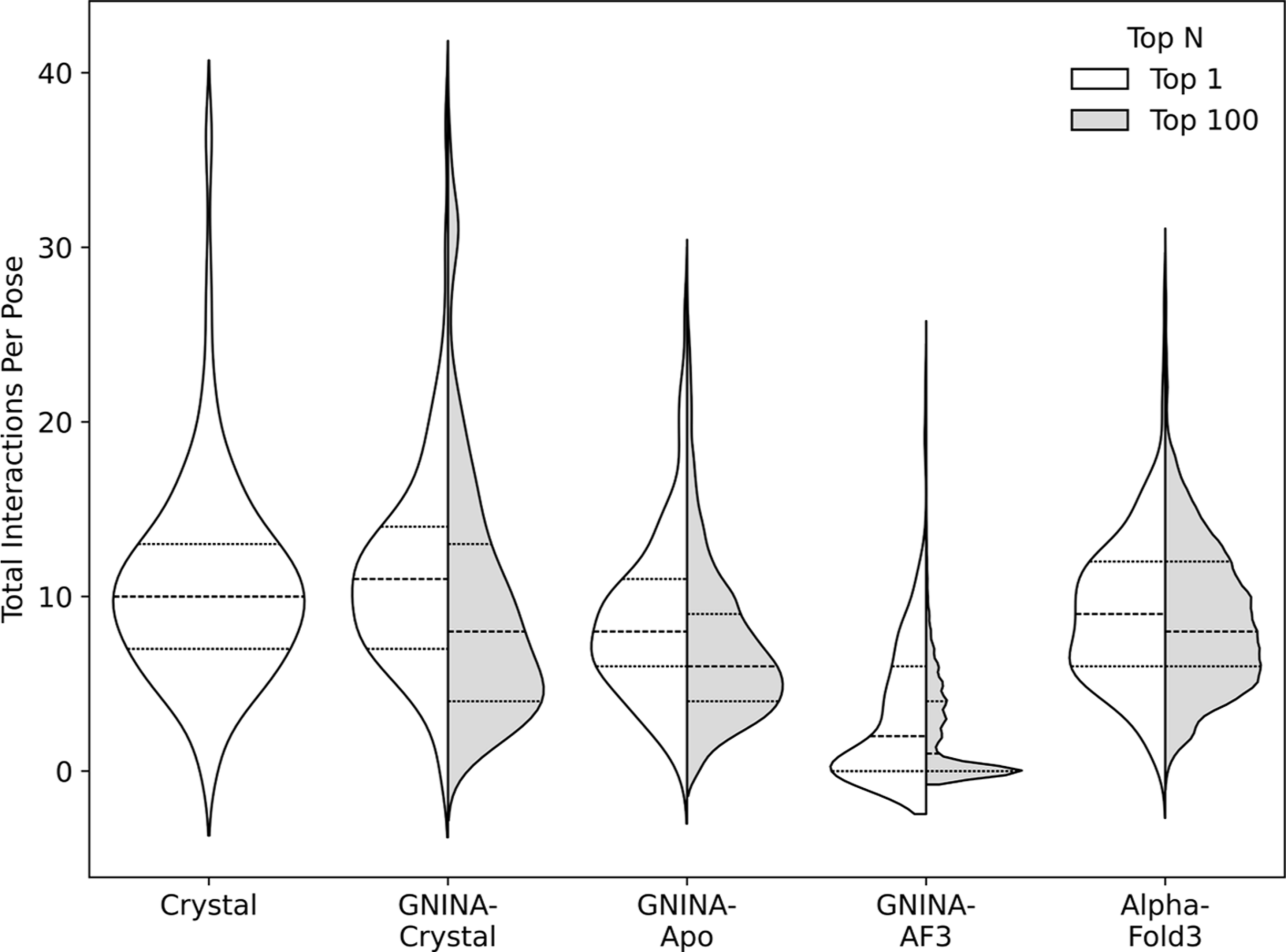
Distribution of PLIP-derived interaction counts per pose across structure sources for the single best pose and top 100 poses. As expected, the single best GNINA-Crystal pose shows the most crystal-like distribution, followed by GNINA-Apo for one pose. The interaction distribution becomes distorted from the crystal reference by use of predicted receptor structures, though AlphaFold3 co-folding appears to recover a more near-crystal distribution than does GNINA-AF3. For 100 poses, distributions broaden as near-crystal interaction distributions are diluted by less favorable poses; AlphaFold3 co-folding is a notable exception, as its poses are broadly similar regardless of rank.

The interaction count distribution of the crystal complexes serves as the reference against which all docking and co-folding conditions were evaluated. For top-ranked poses (top 1), GNINA-Crystal most closely preserved crystal-like interaction patterns. The GNINA-Crystal distribution was the only one statistically indistinguishable from the crystal reference (*p* ≥ 0.105, adjusted). All other conditions differed significantly from crystal but to markedly different degrees. GNINA-AF3 showed the largest divergence (*p* ≤ 10^–56^, adjusted), consistent with the compounded uncertainty introduced by docking into a predicted receptor. Notably, AlphaFold3 co-folding produced interaction distributions significantly different from GNINA-AF3 (*p* ≤ 10^–48^), despite both involving predicted receptor structures of comparable RMSD magnitude (Figure 1). Furthermore, AlphaFold3 distributions were statistically indistinguishable from GNINA-Apo (*p* ≥ 0.115, adjusted). This suggests that co-folding’s compensatory rearrangements of both protein and ligand recover more native-like contacts even when absolute receptor RMSD is large. GNINA-Apo itself differed significantly from the crystal reference (*p* ≤ 10^–5^, adjusted), further supporting the conformational sensitivity of docking to apo receptor structures. For 100-pose ensembles, distributions became increasingly unlike the crystal reference across all sources, as lower-ranked poses with atypical interaction profiles were included.

## Discussion

This study demonstrates that robustness to computationally generated structure inputs is an important and underexplored component of protein–ligand binding affinity prediction (PLBAP) model evaluation. A key finding is that AlphaFold3 co-folding is competitive with—and, for two of five investigated, significantly outperforms—rigid-receptor docking into experimentally resolved apo-receptor conformers. This result directly challenges the assumption that the experimental origin of the receptor is sufficient to guarantee downstream PLBAP quality. Moreover, this establishes AlphaFold3 co-folding as a practical alternative when co-crystal structures are unavailable. Across five independently reproducible PLBAP models, performance consistently degraded under computationally generated inputs relative to crystal structures, reinforcing that crystal-only benchmarks can substantially overestimate expected deployment performance.

### Dependence of Robustness on the Structure Generation Method

The magnitude of degradation was strongly dependent on the structure generation method. Rigid-receptor docking into the holo co-crystal receptor conformer (GNINA-Crystal) supports near-crystal performance, though it is unlikely to be used in deployment settings where co-crystal structures are readily available. Direct comparison between GNINA-Apo and GNINA-Crystal allowed us to isolate the impact of receptor conformational state on PLBAP performance. We found that, despite being experimentally resolved, apo conformers do not reliably recover crystal-level performance and often yield substantial performance losses relative to holo-template docking for CASF-2016. Predicted-receptor docking (GNINA-AF3) introduces additional uncertainty from receptor prediction and can further degrade predictive performance. In contrast, AlphaFold3 co-folding using a single top-ranked pose is competitive against rigid-receptor docking. As shown in Figure 3, this conclusion holds across ensemble sizes, as the PLBAP model’s performance is largely insensitive to the number of AlphaFold3 co-folded poses included. For specific PLBAP models (EHIGN-PLA and GIGN), AlphaFold3 co-folding significantly outperformed GNINA-Apo and GNINA-AF3 (Holm *p* ≤ 0.021). This suggests that joint complex prediction can partially mitigate limitations of docking into perturbed receptor structures. Complementary comparisons using RosettaLigand and Boltz-2 are provided in Supporting Information Appendix S8 and support this finding.

An open question raised by our findings is whether intermediate approaches could close the performance gap between rigid-receptor docking and co-folding. Molecular dynamics (MD)-based workflows can, in principle, recover some receptor flexibility lost under rigid-receptor docking. For example, one could perform docking into an ensemble of receptor conformations sampled via MD or perform postdocking MD relaxation. Given that our PLIP analysis identifies interaction geometry as the primary driver of PLBAP performance rather than global backbone RMSD, methods that better sample local binding site rearrangements may be particularly relevant. Characterizing how MD-based structure generation impacts the PLBAP performance is a practical extension of this work. MD approaches do, however, carry nontrivial costs in expertise and computation, which may constrain their utility in high-throughput screening contexts.

### Multipose Averaging Is Not Universally Beneficial

Pose ensembles are a practical reality for both docking and co-folding, but averaging predictions was not generally useful for CASF-2016. For GNINA-Crystal, averaging across increasing numbers of poses generally degraded the performance. This may imply that lower-ranked poses increasingly include irrelevant ligand placements that dilute the model signal. For GNINA-Apo and GNINA-AF3, there may be a small benefit to averaging over the top few poses; however, this trend was model-dependent and did not recover near-crystal performance. Multipose averaging for AlphaFold3 co-folding ensembles showed marginal performance effects. These findings raise caution against indiscriminate multipose averaging. Further, they suggest that for some structure generation methods, careful pose selection may be more effective than ensemble-based inference.

### Interaction-Level Shifts Help Explain Robustness Losses

RMSD-based perturbation metrics capture global deviation but do not directly reflect the interaction patterns that many PLBAP models implicitly rely on. By profiling all poses with PLIP and comparing interaction count distributions across sources, we find that each structure generation method induces characteristic shifts in hydrogen bonding, hydrophobic contacts, and other interaction classes relative to crystal complexes (see Appendix S9). Notably, predicted-receptor docking and co-folding broaden these distributions, consistent with increased uncertainty in local contact geometry. This interaction-level view provides mechanistic context for the observed performance degradation and highlights why seemingly similar global deviations can distinctly disrupt affinity prediction. Per-complex residual error analyses (Appendix S10) further establish that these effects are population level.

### Limitations of PLBAP Benchmarking

A limitation shared by this study and PLBAP benchmarking more broadly is that all evaluated complexes are confirmed protein–ligand binders. The five models assessed are regression models trained on experimental binding affinities. Even though the range of CASF-2016 affinities spans 10 orders of magnitude (from pico to micromolar),^1^ these five PLBAP models were never tested on pairs for which binding is entirely absent. Whether these models assign systematically lower predicted affinities to nonbinding pairs remains an open question. CASF-2016 does, however, contain complexes with affinities spanning 10 orders of magnitude, capturing very weak and very strong binders (pk range ≈2–12).^1^ The development of a rigorously curated, affinity-relevant negative-control benchmark for PLBAP would be a valuable contribution to the field, though constructing one free of latent binder contamination and target-class bias is a nontrivial undertaking that we view as an important direction for future work. Additionally, all CASF-2016 structures predate the AlphaFold3 training cutoff of September 30, 2021.^1,5^ This means that AlphaFold3 co-folding performance reported here may represent an optimistic upper bound relative to deployment on targets not represented in the AlphaFold3 training data.

### Implications for Model Development and Practical Deployment

A central contribution of this study is to make explicit a benchmark-to-deployment mismatch in PLBAP evaluation. In many publications, PLBAP models are benchmarked on experimentally resolved protein–ligand co-crystal structures, which represent an idealized input condition.^7–10^ In contrast, real users typically apply these models in prospective settings where co-crystal structures are unavailable and computationally generated structures must be used instead.^6^ Thus, supported by our findings here, crystal-only benchmark performance can misrepresent both model readiness for deployment and the level of accuracy end users should expect in practice.

For PLBAP model developers, these findings indicate that benchmarking solely on crystal co-complexes is insufficient for evaluating deployment relevance. If a model is intended for use in high-throughput screening or structure-based discovery pipelines, then its evaluation should include the structure-generation conditions that dominate those workflows, including docking into apo or predicted receptors and co-folded predicted complexes. In other words, robustness to realistic structural perturbations should be treated as a core performance axis, alongside traditional crystal-based benchmark metrics. Reporting performance across multiple deployment-relevant input regimes would provide a more faithful picture of model behavior and improve comparability among PLBAP methods.

For end users, including protein engineers, protein scientists, and drug discovery practitioners, these results highlight the need to interpret published PLBAP performance metrics in the context of input structure type. Performance reported on crystal co-complexes should often be viewed as an optimistic upper bound when the intended workflow relies on computed structures. In practical terms, users should expect that prediction quality may decrease when using co-folded predicted complexes or docked poses in apo/predicted receptors, and they should prefer PLBAP tools that have been explicitly evaluated under input conditions that match their intended use case.

More broadly, the relevant question for PLBAP evaluation is not only how well a model performs on ideal crystal structures but also how well it performs under the structure-generation conditions that define real deployment. Incorporating deployment-relevant computed structures into PLBAP benchmarking and, potentially, into model development and training would help align reported performance with practical utility and support more reliable adoption in real discovery workflows.

## Methods

### Data Set

The CASF-2016 data set (PDBbind core set) was used as the reference benchmark for all evaluations.^1,17^ The CASF-2016 benchmark consists of 285 protein–ligand complexes with experimentally measured binding affinities standardized as p*K* values. All models were evaluated against the same experimental affinity values to ensure consistent cross-model and cross-input comparisons.

### Structure Generation

#### AlphaFold3

Predicted protein–ligand complex structures were generated for each CASF-2016 target using AlphaFold3.^5^ Protein sequence and ligand specifications were derived directly from the CASF-2016 complexes, and multiple sequence alignments were generated using MMseqs2 via ColabFoldAPI. An ensemble of 100 AlphaFold3 co-folding poses were generated per complex (20 seeds, five samples per seed). Full details of AlphaFold3 input preparation, execution, and postprocessing are provided in the Supporting Information Appendix S2. Investigations including Boltz-2 were also performed and are included in the Supporting Information, Appendix S8.

#### GNINA Docking into Crystal Receptors (GNINA-Crystal)

Docked protein–ligand poses were generated using GNINA v1.3.1, a deep-learning-augmented derivative of AutoDock Vina.^4^ Docking was performed using a command-line compatible Python wrapper script executed via SLURM array jobs on GPU nodes. For each CASF-2016 complex, docking boxes were defined using a hybrid ligand- and pocket-based strategy. Box centers were derived from preprocessed pocket coordinates included in the PDBbind core set. GNINA was used to generate up to 1000 poses per complex. The top 100 poses, ranked by GNINA docking score, were retained for downstream affinity prediction. Detailed GNINA input preparation, docking parameters, and postprocessing steps are described in Supporting Information Appendix S4a. Extended investigations were performed with RosettaLigand docking as detailed in the Supporting Information.

#### GNINA Docking into Apo Crystal Receptors (GNINA-Apo)

For targets with experimentally resolved apo receptor conformations, we docked the ligand of interest into the apo receptor structure using the same GNINA protocol as GNINA-Crystal. To ensure comparable binding site localization, the docking region was defined by transferring the crystal binding site frame to the apo conformer via receptor alignment (see Appendix S4b).

#### GNINA Docking into AlphaFold3-Predicted Receptors (GNINA-AF3)

To explore how computationally generated perturbations may impact docking-based predictions, we also performed GNINA docking against the AlphaFold3-predicted receptor structure. For each CASF-2016 target, a receptor-only structure was generated using AlphaFold3 ^5^ from the target protein sequence. AlphaFold3-predicted receptors were converted from CIF to PDB file type with OpenBabel^18^ for docking. GNINA docking was executed using the AlphaFold3-predicted receptor structure and the ligand SDF included in the CASF-2016 data set. Aside from the use of the AlphaFold3-predicted receptor structure and alignment of AlphaFold3 coordinates to the crystal protein coordinates (see Appendix S4c), the same docking workflow was used as in GNINA-Crystal.

### PLBAP Model Selection

For this study, we considered including structure-based protein–ligand binding affinity prediction (PLBAP) models originally published between 2021 and 2024 that reported CASF-2016 p*K* scoring power (18 models).^6–10,19–31^ To be included in the study, we required strict reproducibility of originally reported performance. Independent reproduction of the originally reported CASF-2016 scoring-power Pearson correlation coefficient (PCC) should be reasonably achieved. That is, we must be able to reproduce the originally reported PCC within a 95% confidence interval using 5,000-iteration bootstrapping.^32^ For this study, we investigated 18 models that met the above three criteria. Of these, only nine were end-to-end runnable, and only five met this reproducibility threshold. This reproducibility requirement was imposed to ensure that observed performance differences could be attributed to input structure variation, rather than to undocumented implementation details or environment discrepancies. Of all 18 models initially attempted for reproduction, only five were able to be sufficiently reproduced: Dynaformer,^7^ EGNA,^8^ EHIGN-PLA,^9^ GIGN,^10^ and OnionNet-2.^6^ Pose preparation for all models is described in Appendix S6. Details regarding code sourcing and versioning, pretrained weights, and execution procedures are provided in Supporting Information Appendix S7.

### Evaluation Protocol

For each model and structure source, we computed PCC relative to experimental CASF-2016 affinities. For GNINA docking ensembles, predictions were aggregated across the top *N* poses per complex as the mean value, where *N* ∈ {1, 2, 3, 5, 10, 20, 30, 50, 100} poses. This analysis was used to assess the sensitivity to pose selection and the impact of multipose averaging on predictive performance.

### Computing Environment

All experiments were performed on NVIDA V100 and H200 GPU nodes available through the Michigan State University High Performance Computing Cluster. Full environment specifications, container images, and execution scripts used are documented in the Supporting Information.

## Supplementary Material



## Data Availability

All data generated in this study, including all generated structure poses, predicted binding affinities, and protein–ligand interaction information, are available at 10.5281/zenodo.18701481. All original code is available at https://github.com/WoldringLabMSU/PLBAP_Robustness or in repositories linked in the Supporting Information document.

## Notes

Detailed methodology, complementary results, and extended discussion (PDF)

## Abbreviations

PLBAP: protein–ligand binding affinity prediction
PCC: Pearson correlation coefficient
RMSD: root mean square deviation
AF3: AlphaFold3
PLIP: protein–ligand interaction profiler
