# Supplementary material for: Robustness of Protein–Ligand Binding Affinity Prediction Models to Docked and Predicted Structures
Source: J Chem Inf Model. 2026 Jun 23;66(13):7405–13. doi: 10.1021/acs.jcim.6c00592 (PMC13370857; doi:10.1021/acs.jcim.6c00592)
Supplement: Supplementary file 1 [file ci6c00592_si_001.pdf]

Supplementary Information: Robustness of Protein-Ligand Binding Affinity Prediction Models to Docked and Predicted Structures

Joelle N. Eaves[1,2] and Daniel R. Woldring*[1,2]

[1]Department of Chemical Engineering and Materials Science, Michigan State University, East Lansing, MI, USA
[2]Institute for Quantitative Health Science and Engineering, Michigan State University, East Lansing, MI, USA

*Email: woldring@msu.edu

Appendix S1. RMSD Calculations and Alignment

Alignment Protocol

Alignment of each computational receptor structure to the corresponding crystal receptor was performed with BioPython PairwiseAligner tool.[1] From this alignment, root-mean-squared deviation (RMSD) from the crystal reference structures were computed for each complex and structure source using the MDAnalysis python library.[2]

RMSD Calculation

Protein backbone RMSD was computed using C-alpha atoms (`protein and name CA and chainID X`), where the proper chainID X was determined from the reference crystal to be the chain that contained the binding site. Protein sidechain RMSD was computed on selection `not backbone and not name H*`. Ligand RMSD was computed similarly with the MDAnalysis library from the selection `not element H`. For all RMSD calculations, MDAnalysis rms functions were passed True for arguments `center` and `superimpose`. To ensure consistent comparisons across sources, all structures were preprocessed to ensure residue numbering was consistent between the crystal and computational pose. The exact scripts used to execute these computations are available at https://github.com/WoldringLabMSU/PLBAP_Robustness.

Extended Results

RMSD distributions were summarized using mean, median, and standard deviation in Tables S1 – S3 for protein C-alphas, protein sidechains, and ligand heavy atoms, respectively.

Table S1: Protein C-Alpha RMSD mean, median, and standard deviation from the crystal structure for each structure generation method using the single best poses

Structure Generation Method	Mean RMSD (Å)	Median RMSD (Å)	Standard Deviation (Å)
GNINA-Crystal	0.00	0.00	0.00
Rosetta	0.08	0.03	0.35
GNINA-Apo	5.23	3.07	5.80
GNINA-AF3	5.08	0.80	7.20
AlphaFold3 Co-Folding	5.03	0.65	7.22
Boltz-2 Co-Folding	5.05	0.60	7.21

Table S2: Protein sidechain RMSD mean, median, and standard deviation from the crystal structure for each structure generation method using the single best poses

Structure Generation Method	Mean RMSD (Å)	Median RMSD (Å)	Standard Deviation (Å)
GNINA-Crystal	0.00	0.00	0.00
Rosetta	0.58	0.57	0.27
GNINA-Apo	4.66	3.05	4.89
GNINA-AF3	5.37	1.77	6.39
AlphaFold3 Co-Folding	5.20	1.47	6.51
Boltz-2 Co-Folding	5.16	1.45	6.52

Table S3: Ligand heavy atom RMSD mean, median, and standard deviation from the crystal structure for each structure generation method using the single best poses

Structure Generation Method	Mean RMSD (Å)	Median RMSD (Å)	Standard Deviation (Å)
GNINA-Crystal	3.83	3.63	1.35
Rosetta	3.66	3.51	1.39
GNINA-Apo	3.68	3.62	1.49
GNINA-AF3	3.71	3.59	1.50
AlphaFold3 Co-Folding	3.63	1.44	
Boltz-2 Co-Folding	3.44	3.37	1.51

Appendix S2. AlphaFold3 Inference

Overview

AlphaFold3 (AF3)[3] ligand co-folding was used to generate 100 protein-ligand complex predictions for each CASF-2016 target. AF3 inference was executed using a containerized deployment on GPU nodes from the Michigan State University (MSU) High-Performance Computing Cluster (HPCC). All methods and code used in this study were adapted from our AF3 pipeline: https://github.com/WoldringLabMSU/AlphaFold3-Pipeline.

Input Preparation

For each CASF-2016 complex,

1. Protein sequences were extracted from the CASF-2016 receptor PDB files,[4,5]

2. Ligands were specified using OpenEye SMILES strings reported in the RCSB LigandExpo[6] corresponding to the crystallographic ligands, and

3. AF3 input JSON files were generated from a standardized template.

MSA Generation

Multiple sequence alignments (MSAs) were generated using MMseqs2 via the ColabFold API, executed with `--db-load-mode 2` to support large batch inference.[3] Generated `.a3m` files were moved into per-target directories and referenced directly in the AF3 JSON inputs using automated patching scripts.

Execution

AF3 inference was executed using a Singularity image preinstalled on the MSU HPCC. Outputs poses were ranked using the default output ranking csv from AlphaFold3.

Postprocessing

AF3 outputs were converted to standard PDB format, validated for missing atoms or malformed coordinates, and renamed by PDB ID.

Appendix S3. Boltz-2 Inference

Overview

Boltz-2[7] was used to generate a single protein-ligand complex per CASF-2016 target using Boltzmann-weighted AlphaFold3 inference. All methods and code used in this study were adapted from our Boltz-2 pipeline: https://github.com/WoldringLabMSU/Boltz-2_Pipeline.

Input Preparation

To isolate the effect of Boltz-2 sampling, protein sequences, ligand SMILES, and MSAs were identical to those used for AF3. Boltz-2 YAML configuration files were generated programmatically per target.

Execution

Boltz-2 inference was executed using the official Boltz-2 repository:[7] https://github.com/jwohlwend/boltz.

Postprocessing

As was done for the AF3 outputs, Boltz-2 outputs were converted to standard PDB format, validated for missing atoms or malformed coordinates, and renamed by PDB ID.

Appendix S4. GNINA Docking

Appendix S4.A. GNINA Docking into Crystal Receptor (GNINA-Crystal)

Overview

GNINA v1.3.1[8] was used to generated 3D docked protein-ligand poses for all CASF-2016 complexes. Docking was performed using a CLI-compatible python wrapper script executed via SLURM array jobs on GPU nodes from the MSU HPCC. All methods and code used in this study was adapted from our GNINA docking pipeline (https://github.com/WoldringLabMSU/GNINA-Docking-Pipeline) and the official GNINA repository (https://github.com/gnina/gnina).[8]

Input Preparation

For each CASF-2016 complex,

1. Protein structures were extracted from the CASF-2016 receptor PDB files,[4]
2. extracted from the original crystal CASF-2016 PDB files and converted to `.sdf` format using Open-Babel,[9]
3. Binding pockets were defined using preprocessed `.pdb` files from PDBbind[5] containing only the pocket residues, and
4. The docking box center was computed from the pocket coordinates and the box size was estimated using a hybrid ligand- and pocket-based method detailed in the `docking_utils.py` script available on our GitHub.

Execution

GNINA was executed inside a Singularity container with GPU acceleration enabled. The receptor and ligand files were converted to `.pdbqt` format prior to docking. For each complex, GNINA produced a multi-pose `.pdbqt` file with up to 1,000 poses. Docking jobs were parallelized using SLURM array jobs.

Postprocessing

Output `.pdbqt` files were split into per-pose `.pdb` files using OpenBabel[9] and the top 100 poses of each complex were retained.

Appendix S4.B. GNINA Docking into Apo Crystal Receptor (GNINA-Apo)

Overview

To isolate the effect of receptor conformational state on downstream PLBAP performance, GNINA docking was performed against experimentally resolved apo receptor conformers when available.

Input Preparation

Apo receptor structures were obtained from the PDB and converted to PDB format as needed. Each apo receptor was aligned to its corresponding holo (co-crystal) receptor to transfer the binding-site coordinate frame and ensure consistent docking-box localization. Ligand inputs were identical to GNINA-Crystal (ligand SDF derived from the CASF-2016 complex).

Docking Box Localization

The docking box center and size were defined by transferring the crystal binding-site definition to the apo conformer after alignment. This strategy avoids confounding box-placement error with receptor conformational differences.

Execution and Postprocessing

GNINA execution and postprocessing followed the same protocol as GNINA-Crystal. Up to 1,000 poses were generated per complex and the top 100 poses ranked by GNINA score were retained for downstream evaluation.

Appendix S4.C. GNINA Docking into AlphaFold3-Predicted Receptor (GNINA-AF3)

Overview

To evaluate the effect of receptor structural deviation of PLBAP performance, receptor-only predicted structures were generated via AlphaFold3 in a protein-only mode. GNINA docking was performed using receptor-only AlphaFold3 predicted structures as docking receptors (GNINA-AF3).

Protein-only AlphaFold3 Execution

For each target sequence, a protein-only JSON file was created from the standardized template (`AF3.json`). AlphaFold3 inference was, as described in Appendix S1, executed via Singularity using `run_alphafold.py`. Receptor-only AF3 outputs were stored per target for downstream docking.

AlphaFold3 Mode Alignment and Box Localization

Coordinate frame mismatches between AlphaFold3-predicted receptors and the corresponding crystal complex, from which the ligand input for GNINA docking was parsed. Thus, we enabled a specific `--af3` mode in which the AlphaFold3 receptor is aligned to the corresponding crystal receptor and the crystal ligand coordinates are transformed accordingly. This provides an approximate transformed binding-site from which the ligand coordinates are used to compute a docking box center and boundaries.

Execution and Postprocessing

GNINA execution and postprocessing followed the same protocol as GNINA-Crystal. The top 100 poses were retained per complex.

Appendix S6. Pose Preparation for PLBAP Input

Poses generated by AF3 and Boltz-2 (.cif file type) were converted to .pdb format using OpenBabel.[9] Each PDB complex was then split into separate ligand and protein files (PDB format). Ligand files were further converted into MOL2 and SDF file types with OpenBabel to ensure appropriate input type was readily available for input into downstream PLBAP models. Example code for performing conversions for each input type (GNINA, AF3, etc) is available at https://github.com/WoldringLabMSU/PLBAP_Robustness.

Appendix S7. PLBAP Model Implementation

Code Source and Versioning

For each model, two repositories are referenced. The original repository is the official code repository of the original publication. It is owned and maintained by the model authors and corresponds to the published implementation. The study fork is the repository used to execute the large-scale benchmarking of this work.

The study forks preserve the original model architectures and trained parameters, while incorporating only minimal modifications required for compatibility with current software dependencies, batch processing of multiple pose sources, standardized input/output handing across models, and time logging. No architectural changes or retraining were performed for any model. All forks are publicly accessible and linked in Table S4.

Input Preparation and Compatibility

Each model requires a distinct input representation, including grid-based, graph-based, or geometric features. For consistency, all models were executing using their native pipelines with only the following adjustments:

1. Input file paths were parameterized, when possible, to support batch evaluation across multiple structure sources.

Table S4: GitHub repositories published alongside official models and respective forks applied in this study

Model	Original Repository	Study Fork
Dynaformer	https://github.com/Minys233/Dynaformer	https://github.com/jeavesj/Dynaformer
EGNA	https://github.com/chunqiux/EGNA	https://github.com/jeavesj/EGNA
EHIGN-PLA	https://github.com/guaguabujianle/EHIGN_PLA	https://github.com/jeavesj/EHIGN_PLA
GIGN	https://github.com/guaguabujianle/GIGN	https://github.com/jeavesj/GIGN
OnionNet-2	https://github.com/zchwang/OnionNet-2	https://github.com/jeavesj/OnionNet-2

2. Docked and predicted structures were converted to the expected input format without altering atomic coordinates.

No modifications were made to feature definitions, normalization schemes, or target transformations beyond those executed by the original authors' pipelines.

Model Execution

All models were executed in inference-only mode (i.e., no re-training) using author-provided inference/evaluation scripts or minimally adapted wrappers.

Output Organization

All model predictions were converted to a standardized CSV format consisting of columns described in Table S5.

Table S5: Standardized PLBAP report format with column names and descriptions

Column Name	Description
pdbid	Unique identifier for each complex (lowercase)
Source	Crystal, GNINA-Crystal, GNINA-Apo, GNINA-AF3, Rosetta, AlphaFold3-Cofold, or Boltz-2
Model	Dynaformer, EGNA, EHIGN_PLA, GIGN, or OnionNet-2
pose	Pose ID with leading zeros to four-places (e.g., 0001, 0002) (dtype: str)
pK_true	Experimental pK value from PDBbind (dtype: float)
pK_predicted	Predicted pK value for the given sample (dtype: float)

Appendix S8. Extended Analysis of Boltz-2 and RosettaLigand

Overview

The main text focuses on the most common deployment decision points for PLBAP: rigid-receptor docking into a holo (co-crystal-derived) receptor (GNINA-Crystal), rigid-receptor docking into an experimentally resolved apo conformer (GNINA-Apo), rigid-receptor docking into a predicted receptor (GNINA-AF3), and full complex co-folding (AlphaFold3). Here, we report supplemental analyses for two additional baselines, RosettaLigand docking and Boltz-2 co-folding, to assess whether the principal robustness trends observed in the main text generalize across alternative structure-generation paradigms.

RosettaLigand provides a semi-flexible docking baseline (explicit ligand conformer sampling with local receptor sidechain flexibility), whereas Boltz-2 provides an alternative co-folding baseline that returns a single predicted complex per target under Boltzmann-weighted sampling. We emphasize that these results are not intended to establish a new *best* structure generator; rather, they contextualize the main-text findings by showing that (i) docking approaches that preserve holo receptor geometry tend to be the most compatible non-crystal inputs for current PLBAP models, and (ii) predicted-complex paradigms (co-folding) remain meaningfully shifted from the crystal training/benchmark regime for most PLBAP pipelines.

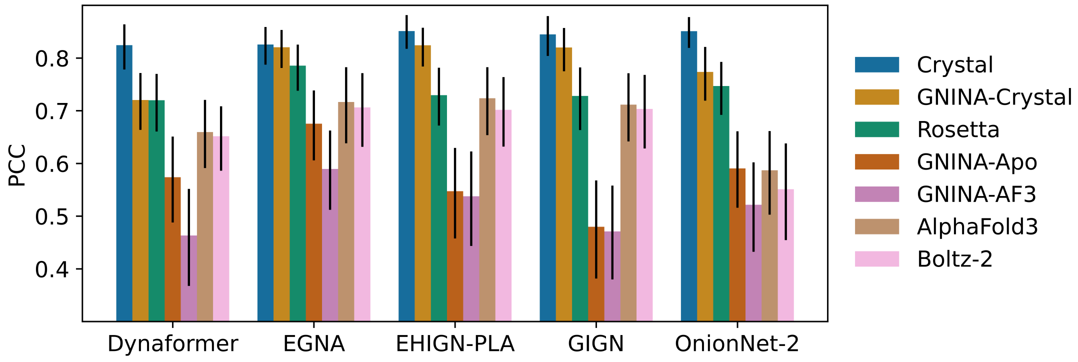

Figure S1: Performance represented as Pearson correlation coefficient (PCC) for the top pose from each structure source across models. Extended analysis includes RosettaLigand Docking to the holo crystal conformer, which follows similar trends to GNINA-Crystal, and Boltz-2 co-folding that follows similar trends to AlphaFold3 co-folding.

RosettaLigand docking

Structural perturbations relative to crystal

RosettaLigand docking was performed using crystal receptor structures as input (Appendix S5). Consistent with this design, receptor backbone deviations relative to the crystal reference were near-zero (Table S1), while sidechain RMSD reflected modest local adjustments (Table S2). Ligand RMSD values were similar in magnitude to GNINA-Crystal (Table S3), indicating that the dominant perturbation remains ligand placement under a largely fixed holo receptor coordinate frame. Together, these observations place RosettaLigand in the same overall perturbation regime as GNINA-Crystal (holo-template docking), with the key difference that RosettaLigand permits limited receptor flexibility.

PLBAP performance and relationship to GNINA-Crystal

Across the evaluated PLBAP models, RosettaLigand-based inputs yielded performance that was broadly consistent with the main-text conclusion that preserving holo receptor geometry is critical for minimizing performance degradation. In practice, RosettaLigand behaved similarly to GNINA-Crystal in that both methods avoid large receptor backbone perturbations and therefore maintain a closer match to crystal-like input distributions than predicted-receptor conditions (Figure S1). Any remaining performance gap relative to crystal inputs is expected to arise primarily from ligand placement error and, secondarily, from the sidechain adjustments introduced by semi-flexible docking (Table S2). These results support the main-text interpretation that receptor backbone perturbation is a dominant driver of robustness loss, while local sidechain changes and ligand misplacement contribute additional, method-dependent effects.

Effect of pose-ensemble aggregation

RosettaLigand produces a large pose ensemble per target (1,000 poses generated; top 100 retained; Appendix S5), enabling explicit assessment of multi-pose averaging. In contrast to GNINA-Crystal—where averaging across increasing numbers of poses often diluted signal by incorporating progressively poorer ligand placements—RosettaLigand ensembles can benefit from modest aggregation in some settings (Figure S2). This is consistent with the intuition that local sidechain accommodation may stabilize a subset of plausible interaction geometries across poses. However, these benefits are not universal across models and ensemble sizes, reinforcing the main-text message that pose-ensemble strategies should be treated as source-dependent rather than assumed to be broadly beneficial.

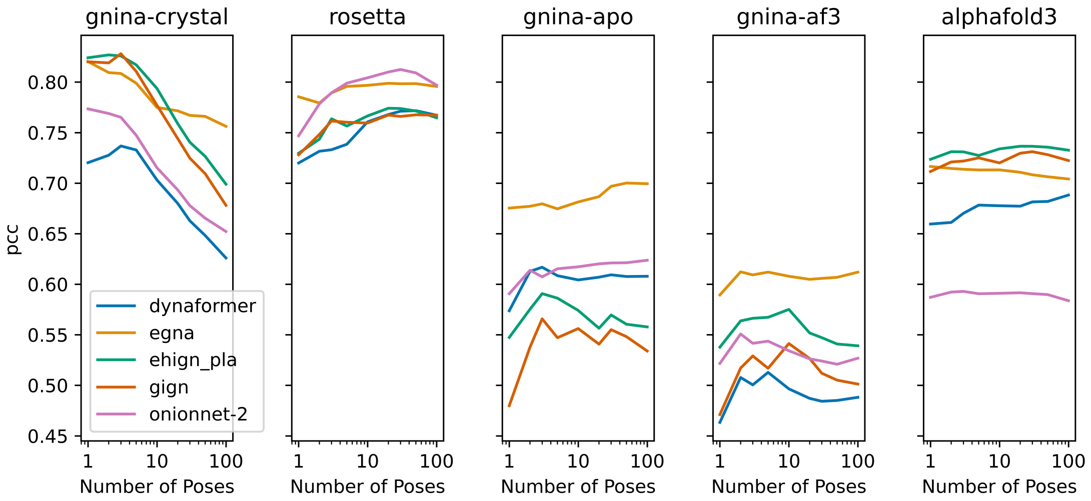

Figure S2: Multipose averaging shows distinct improvement in predictive performance for the RosettaLigand condition, in contrast to the GNINA-Crystal trends. This is likely explained by the local sidechain flexibility and extended ligand conformer sampling offered by RosettaLigand.

Boltz-2 co-folding

Structural perturbations relative to crystal

Boltz-2 co-folding (Appendix S3) produced receptor backbone and sidechain deviations comparable in magnitude to AlphaFold3 co-folding (Tables S1–S2), reflecting that both methods generate predicted complex conformations rather than preserving the experimentally resolved holo receptor geometry. While ligand RMSD may be modestly reduced on average for Boltz-2 relative to some docking-based conditions (Table S3), the dominant distribution shift arises from the predicted receptor geometry and its coupled protein–ligand arrangement. Thus, Boltz-2 represents an alternative predicted-complex regime that is expected to stress-test PLBAP robustness similarly to AlphaFold3 co-folding.

PLBAP performance and relationship to AlphaFold3 co-folding

Boltz-2-based inputs exhibited the same qualitative pattern observed for AlphaFold3 co-folding in the main text: performance is degraded relative to crystal inputs and generally reflects a mismatch between the structural priors learned by PLBAP models (typically trained and validated primarily on crystal complexes) and the statistical properties of predicted complexes. Importantly, this does not imply that Boltz-2 (or AlphaFold3) complexes are intrinsically poor physical hypotheses. Rather, it indicates that current PLBAP pipelines are not uniformly robust to the interaction-geometry and receptor-conformation shifts induced by modern co-folding paradigms.

Because Boltz-2 yields a single predicted complex per target in our protocol (Appendix S3), it does not provide a direct analogue of the 100-pose AlphaFold3 ensemble used for the main-text co-folding analyses. As a result, Boltz-2 should be interpreted as a *single-shot* predicted-complex baseline, whereas AlphaFold3 results quantify how extensive sampling impacts robustness under co-folding.

Interaction-level comparison using PLIP

To complement RMSD-based perturbation metrics, we profiled poses with PLIP and summarized per-pose interaction counts by interaction class (Appendix S9). Consistent with the main-text analysis, docking-based sources that preserve holo receptor geometry (GNINA-Crystal and RosettaLigand) tend to produce interaction-count distributions that more closely resemble crystal references, particularly for high-frequency interaction classes (e.g., hydrophobic contacts and hydrogen bonds). In contrast, predicted-

complex paradigms (AlphaFold3 co-folding and Boltz-2) broaden interaction-count distributions and shift the prevalence of specific interaction classes, reflecting increased uncertainty in local contact geometry and binding-site configuration. These interaction-level shifts provide mechanistic context for why PLBAP performance can degrade substantially even when global RMSD differences appear comparable across predicted-receptor conditions.

Conclusions relative to the main text

The supplemental RosettaLigand and Boltz-2 analyses reinforce three core conclusions from the main text:

1. **Preserving holo receptor geometry is strongly associated with improved robustness.** RosettaLigand (semi-flexible docking into crystal receptors) behaves most similarly to GNINA-Crystal and remains substantially closer to the crystal benchmark regime than predicted-receptor conditions.

2. **Predicted-complex regimes remain challenging for current PLBAP pipelines.** Boltz-2 co-folding shows robustness limitations consistent with AlphaFold3 co-folding, supporting the interpretation that receptor- and interaction-geometry shifts, rather than the specific co-folding implementation, drive much of the observed degradation.

3. **Ensemble strategies are source-dependent.** RosettaLigand ensembles can exhibit modest gains from limited aggregation, whereas GNINA-Crystal aggregation often degrades performance. This underscores that pose-ensemble aggregation should be selected and justified based on the structure source and its error modes.

Appendix S9. Extended Protein-Ligand Interaction Distribution Data

For each pose, either crystal or computationally generated, we extracted all protein-ligand interaction information using the Protein-Ligand Interaction Profiler (PLIP).[10] To supplement Figure 4 in the main text, we provide a breakdown by interaction type in Figure S3.

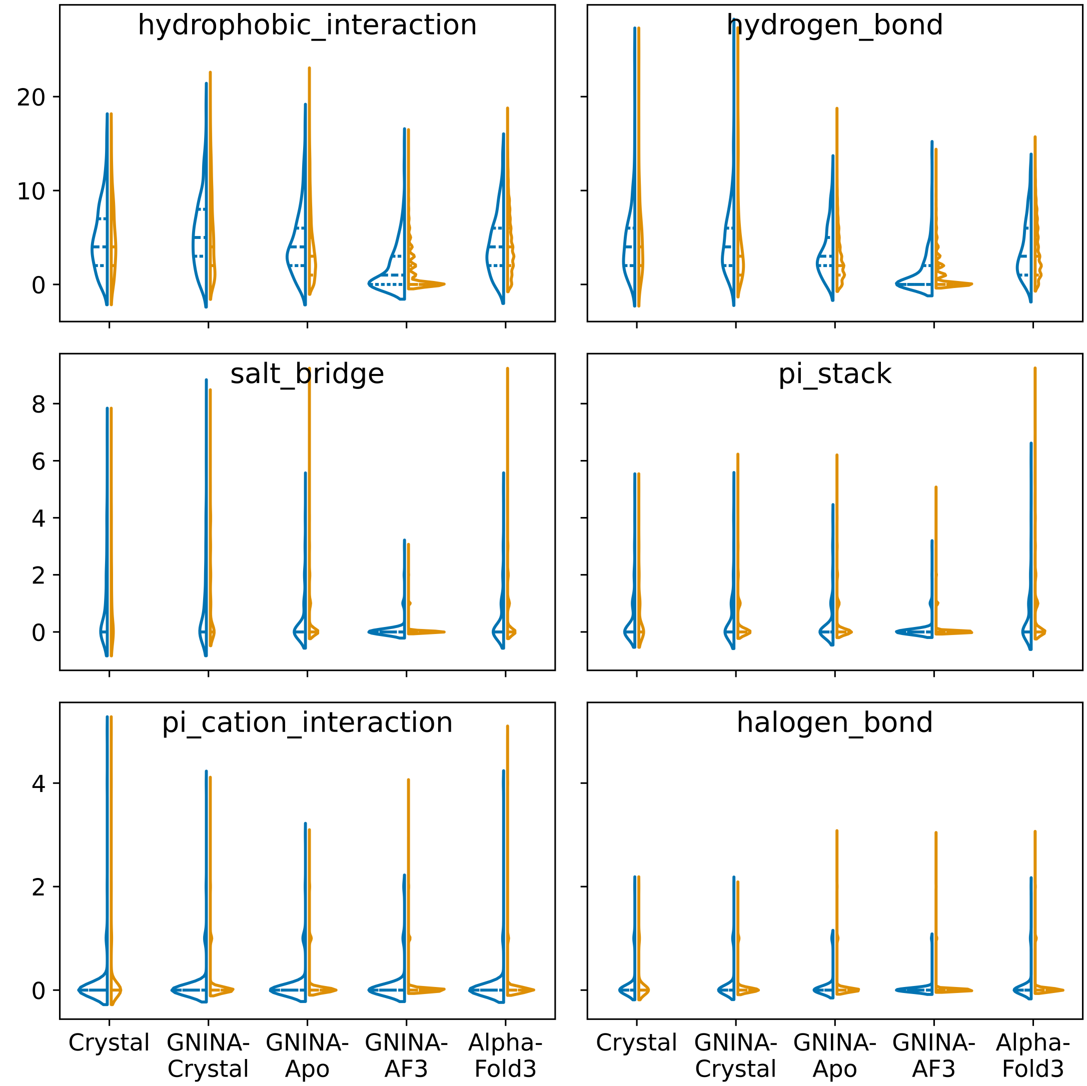

Figure S3: Protein-ligand interaction count distributions, by interaction type, for the top 1 and 100 poses from each structure generation method.

Appendix S10. Interaction Geometry and AlphaFold3 Confidence as Predictors of PLBAP Error

Experimental Affinity vs. AlphaFold3 Confidence

To complement the aggregate PCC-based analysis in the main text and Appendix S9, we investigated explanations of per-complex affinity prediction error. First, we investigated the correlation between two common AlphaFold3 confidence metrics with experimental binding affinity for the best AlphaFold3 co-folding pose of each complex : ranking score and inter-chain predicted aligned error (PAE).[3]

The AlphaFold3 ranking score (`ranking_score`) provides a composite score computed as $0.8 * iPTM + 0.2 * pTM$, where ipTM is the inter-chain predicted template modeling score and pTM is the predicted template modeling score.[3] The composite ranking score has been considered preferable to either independent term.[11] Figure S4 shows the relationship between experimental affinity and AlphaFold3 ranking score. The Spearman correlation ($\rho = +0.12$) indicates a slight positive correlation that is weakly significant (p=0.04).

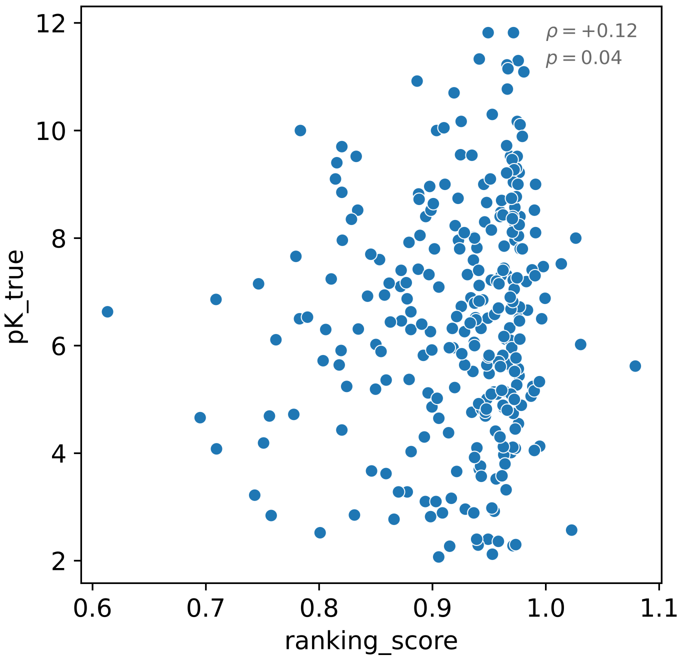

Figure S4: Per-complex experimental affinity as pK verus AlphaFold3 ranking score for the best AlphaFold3 co-folding pose.

We also investigated this correlation for inter-chain PAE, which captures AlphaFold3 confidence in the relative positioning of the protein and ligand chains to one another.[3] Figure S5 shows this relationship, which has a significantly negative correlation ($\rho = -0.30$, $p < 2e-7$). This indicates that mean inter-chain PAE may be more informative for protein-ligand affinity than ranking score.

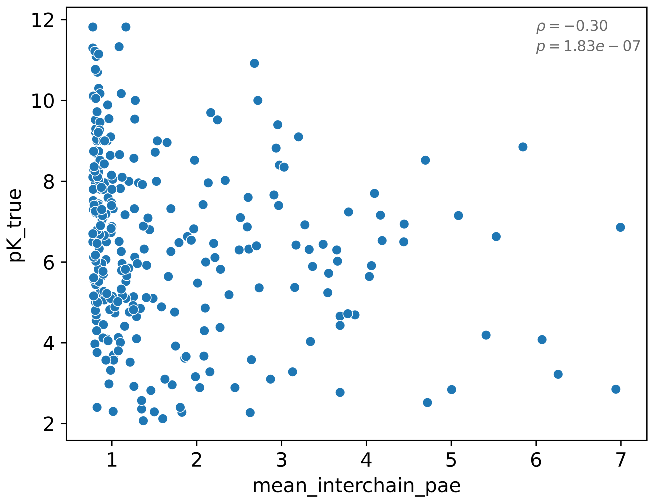

Figure S5: Per-complex experimental affinity as pK verus AlphaFold3 inter-chain predicted aligned error (PAE) for the best AlphaFold3 co-folding pose.

For each structure source and PLBAP model, we computed the per-complex residual ($pK_{pred} - pK_{true}$) for the single best-ranked pose and plotted it against PLIP-derived interaction counts (by type and total), AF3 inter-chain PAE, and AF3 ranking score. Spearman correlations between each of these variables and absolute residual magnitude are annotated in each panel; asterisks denote $p < 0.05$.

These analyses show that per-complex prediction error is not strongly or consistently predicted by any single interaction-geometry or confidence metric across models and structure sources. Most Spearman correlations are near zero and non-significant. Where significant associations are observed, the are isolated to a specific model-source combination rather than any general trend. This suggests that aggregate performance differences across structure sources (Figure 2, main text) arise from more nuanced interaction profile shifts (Figure 4, main text).

AlphaFold3 Confidence vs. Prediction Error

Figure S6 shows the per-complex prediction residual for each PLBAP model as a function of the AlphaFold3 ranking score for the single best AlphaFold3 co-folding pose of each complex. No significant trend was observed. This likely reflects AlphaFold3's uniformly high confidence for targets within its training data, which include CASF-2016.[3] Whether the ranking score is predictive of PLBAP performance for genuinely novel targets, for which confidence scores may span a broader range, remains an open question.

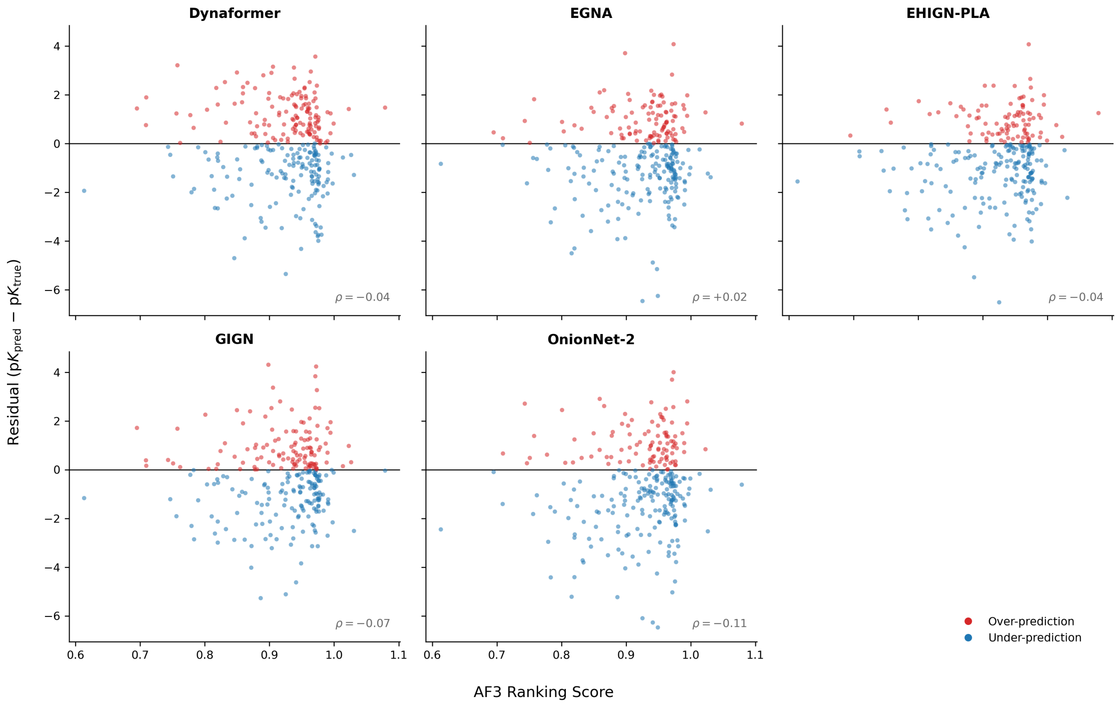

Figure S6: Per-complex prediction residual ($pK_{pred} - pK_{true}$) as a function of the AlphaFold3 ranking score for the single best AlphaFold3 co-folding pose. Spearman ρ is annotated in each panel; no significant associations were observed.

Figure S7 shows a similar analysis for the best AlphaFold3 co-folding pose per complex, instead evaluating by mean inter-chain PAE rather than ranking score. A marginally significant positive association was observed for OnionNet-2 (Spearman $\rho = +0.12$, $p < 0.05$), indicating that complexes with higher inter-chain PAE (lower placement confidence) tend to have larger absolute prediction errors. No significant associations were observed for the other four models. The overall weakness in this trend, again, reflects the uniformly high AlphaFold3 confidence for CASF-2016 targets.

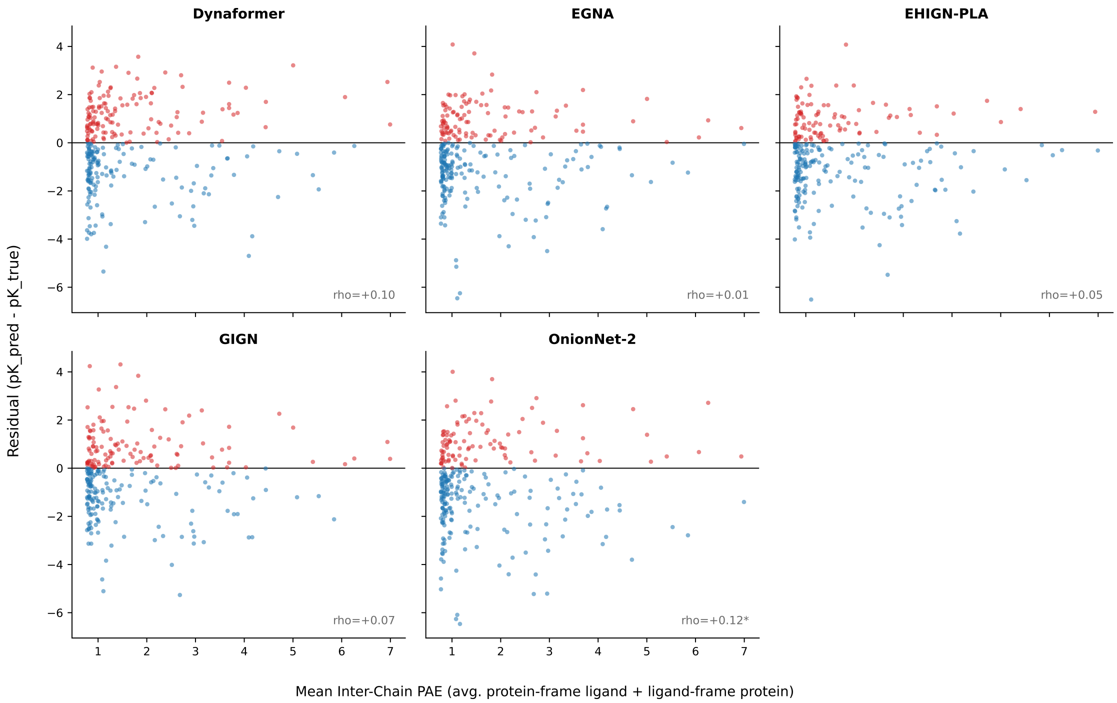

Figure S7: Per-complex prediction residual ($pK_{pred} - pK_{true}$) as a function of the AlphaFold3 mean inter-chain PAE for the single best AlphaFold3 co-folding pose. Spearman ρ is annotated in each panel. Only OnionNet-2 was observed to have a significant association.

Protein-Ligand Interaction Counts vs. Prediction Error

Figures S8-S14 show per-complex prediction residuals as a function of PLIP-derived interaction counts for each interaction type, and for total interactions, across all five structure sources (rows) and all five PLBAP models (columns). We describe the results by interaction type below.

Total Interaction Counts

The total interaction count showed the most consistent associations with prediction error across the dataset (see Figure S8). For GNINA-Apo, significant negative correlations were observed for EHIGN-PLA ($\rho = -0.12^*$) and OnionNet-2 ($\rho = -0.17^*$). This indicates that GNINA-Apo poses with more total interactions actually are predicted more accurately by these models. No significant associations were observed for any other source-model combinations.

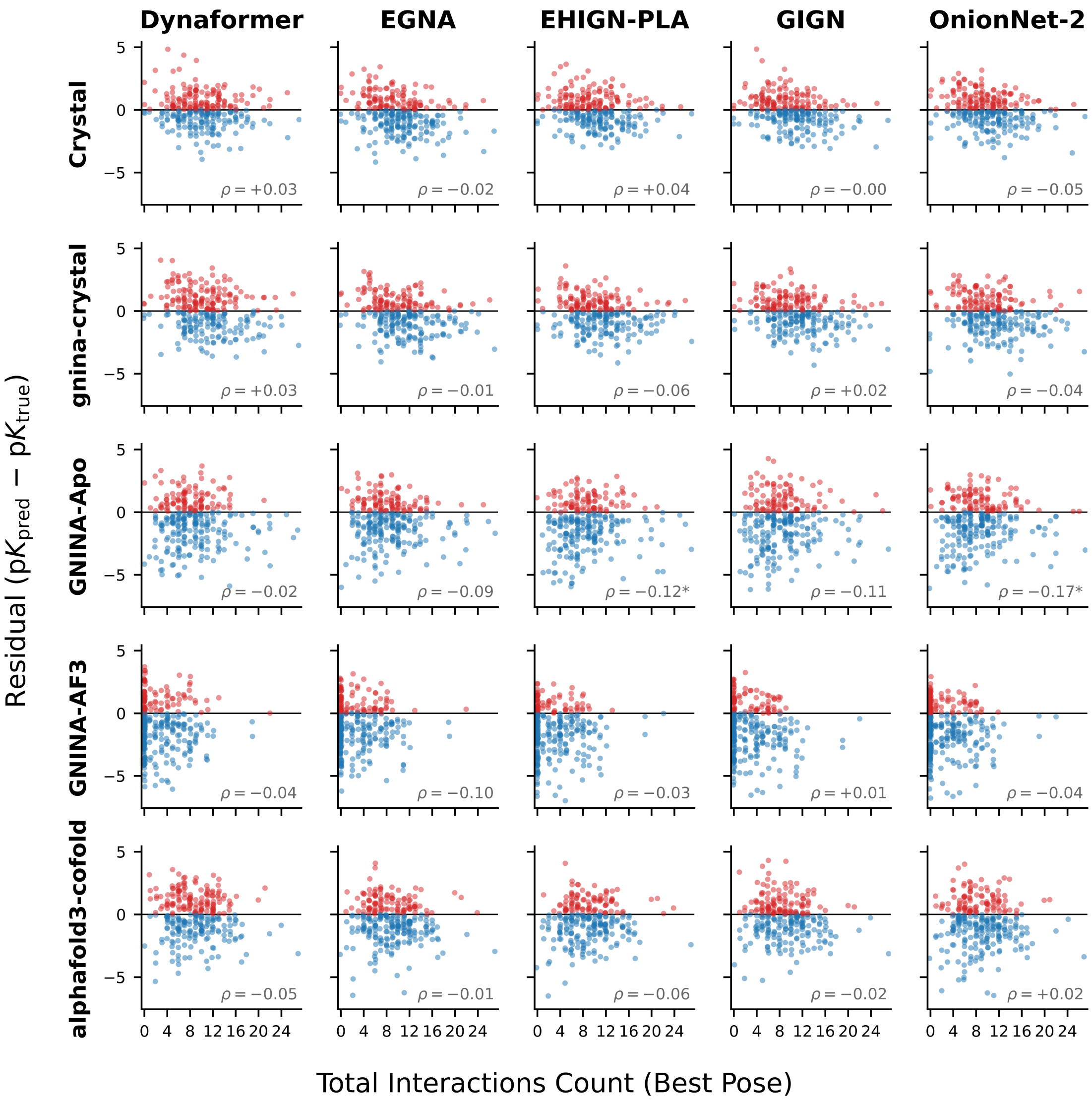

Figure S8: Per-complex prediction residual vs. total interaction counts (PLIP, best pose) across all structure sources (rows) and PLBAP models (columns). Spearman ρ between count and absolute residual is annotated; asterisks denote $p < 0.05$.

Hydrophobic Interactions

Hydrophobic contacts are the most frequent interaction type across all sources. A significant negative association was observed for EHIGN-PLA under GNINA-Crystal ($\rho = -0.13^*$). Associations for other model-source combinations were weak and nonsignificant (see Figure S9).

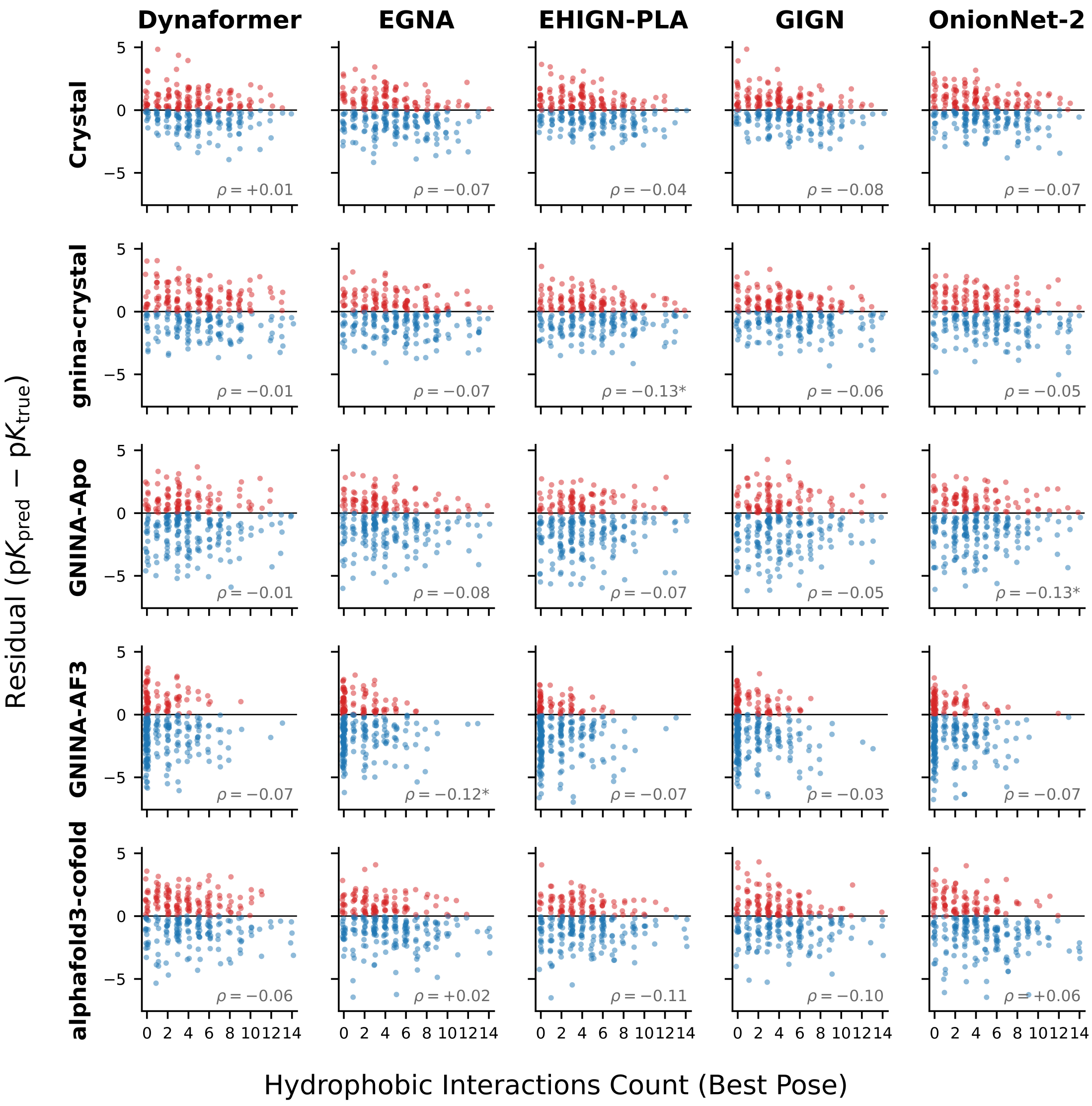

Figure S9: Per-complex prediction residual vs. hydrophobic interaction counts (PLIP, best pose) across all structure sources (rows) and PLBAP models (columns). Spearman ρ between count and absolute residual is annotated; asterisks denote $p < 0.05$.

Hydrogen Bonds

A significant positive association was observed for GIGN under Crystal inputs ($\rho = +0.12^*$) and GNINA-Crystal ($\rho = +0.13^*$) (see Figure S10). This indicates that complexes with more hydrogen bonds show slightly larger prediction error for these models and sources. This may reflect GIGN's specific sensitivity to hydrogen bond geometry, though the effect size is small.

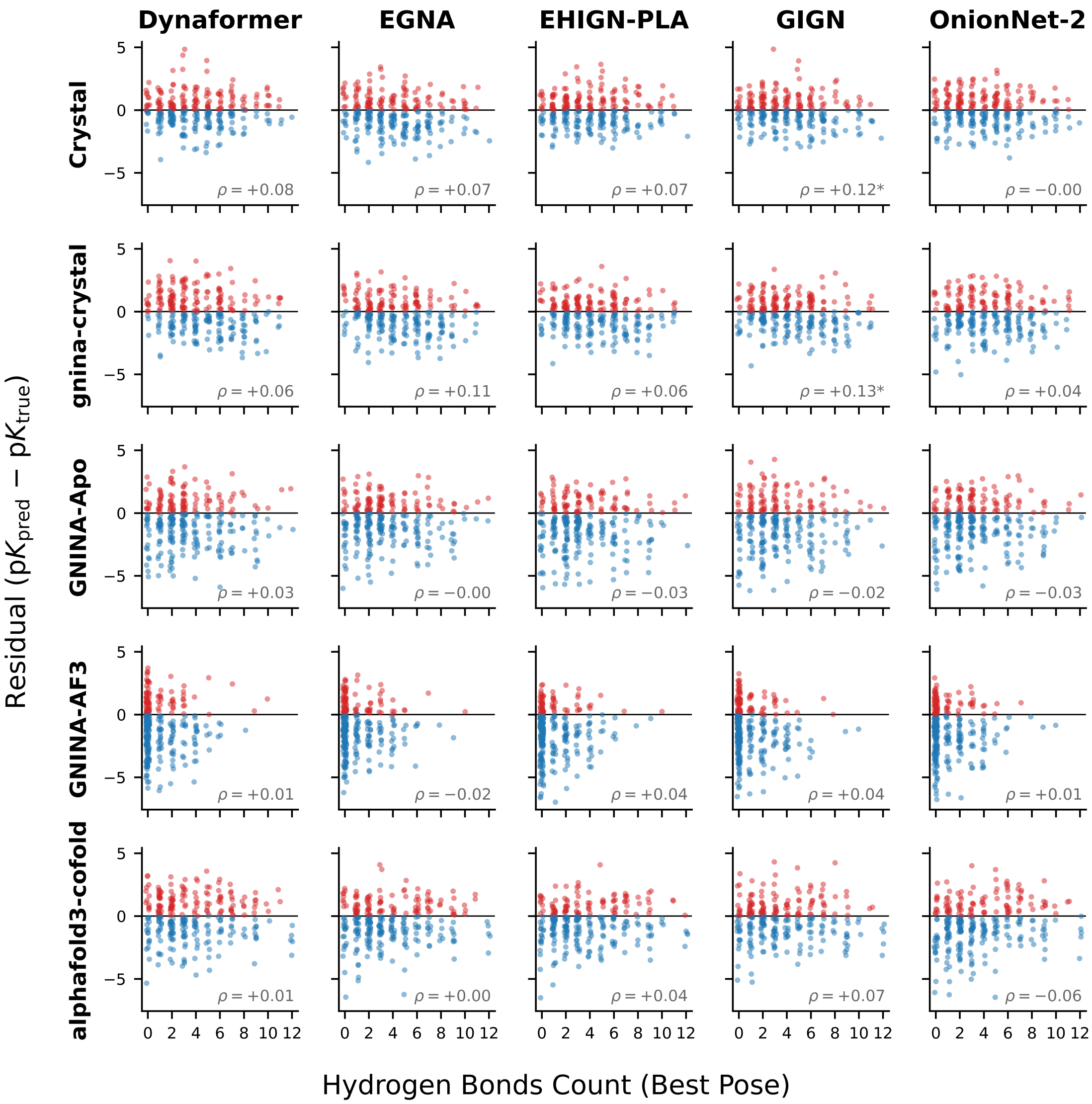

Figure S10: Per-complex prediction residual vs. hydrogen bond (PLIP, best pose) across all structure sources (rows) and PLBAP models (columns). Spearman ρ between count and absolute residual is annotated; asterisks denote $p < 0.05$.

Pi-Stacking

Significant positive associations were observed for EHIGN-PLA and OnionNet-2 under GNINA-AF3 ($\rho = +0.16^*$ for both). This indicates that GNINA-AF3 poses with more pi-stacking contacts tend to have larger prediction errors for these models. No significant associations were observed for other model-source pairs, including AlphaFold3 co-folding (see Figure S11). It is possible that this reflects that co-folding resolves pi-stacking contacts more accurately, also supported by Figure S3.

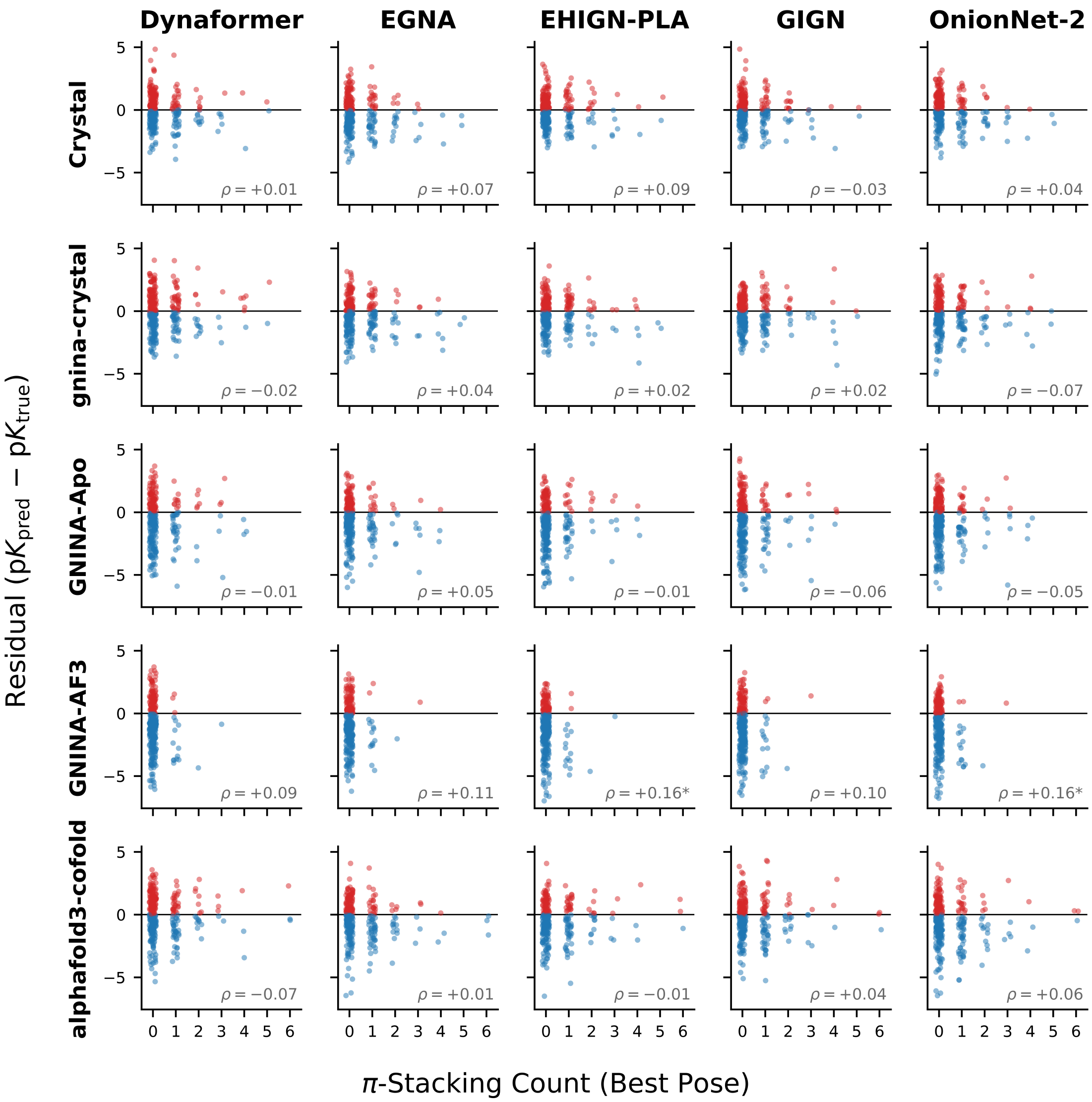

Figure S11: Per-complex prediction residual vs. pi-stacking contacts (PLIP, best pose) across all structure sources (rows) and PLBAP models (columns). Spearman ρ between count and absolute residual is annotated; asterisks denote $p < 0.05$.

Pi-Cation Interactions

A significant negative association was observed for EGNA under GNINA-Apo ($\rho = -0.15^*$). No consistent pattern was observed across other model–source combinations. See Figure S12.

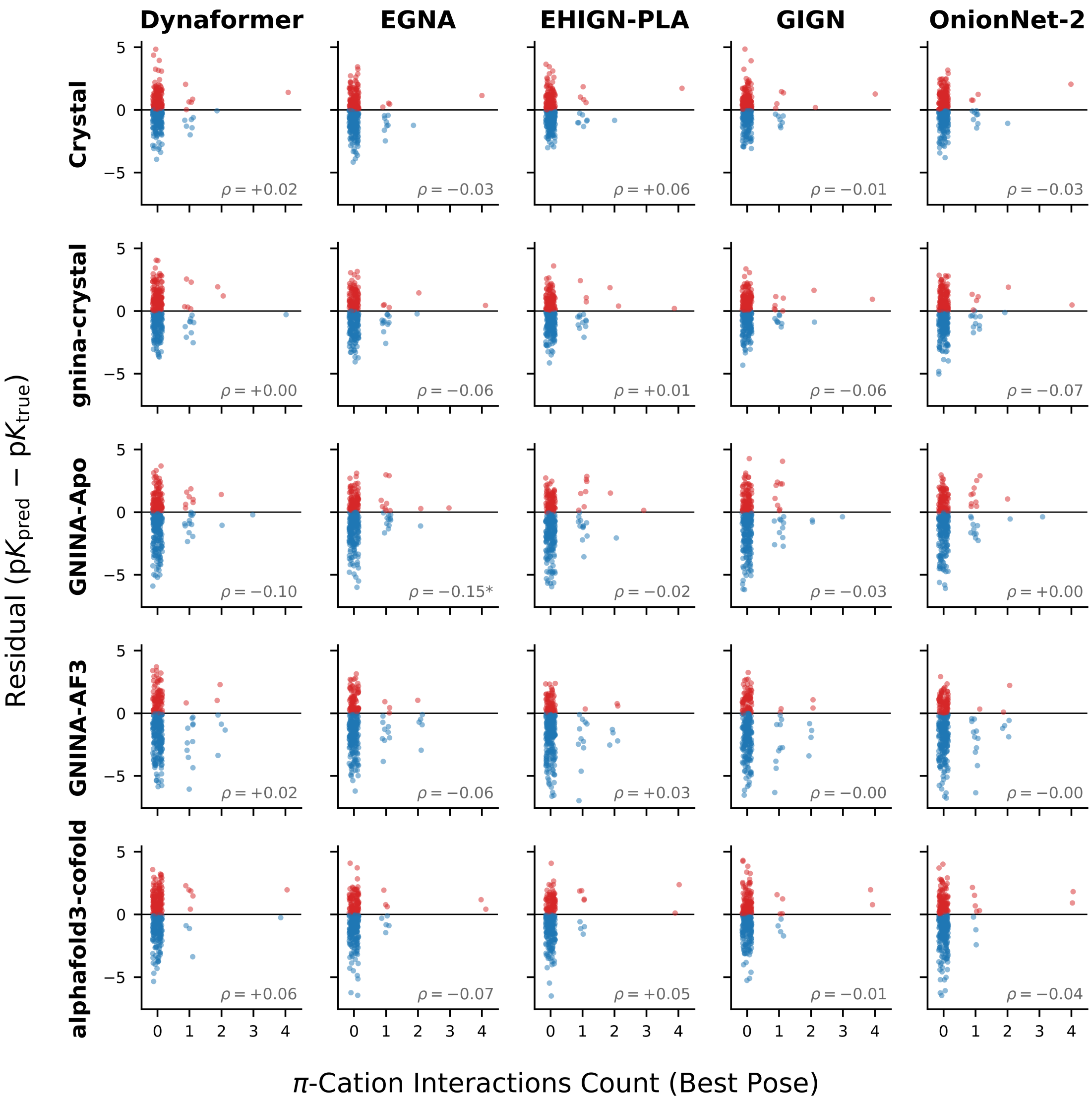

Figure S12: Per-complex prediction residual vs. pi-cation interactions (PLIP, best pose) across all structure sources (rows) and PLBAP models (columns). Spearman ρ between count and absolute residual is annotated; asterisks denote $p < 0.05$.

Salt Bridges

No significant associations were observed for any model–source combination. Salt bridges are relatively infrequent across CASF-2016 and their distribution is sparse. See Figure S13.

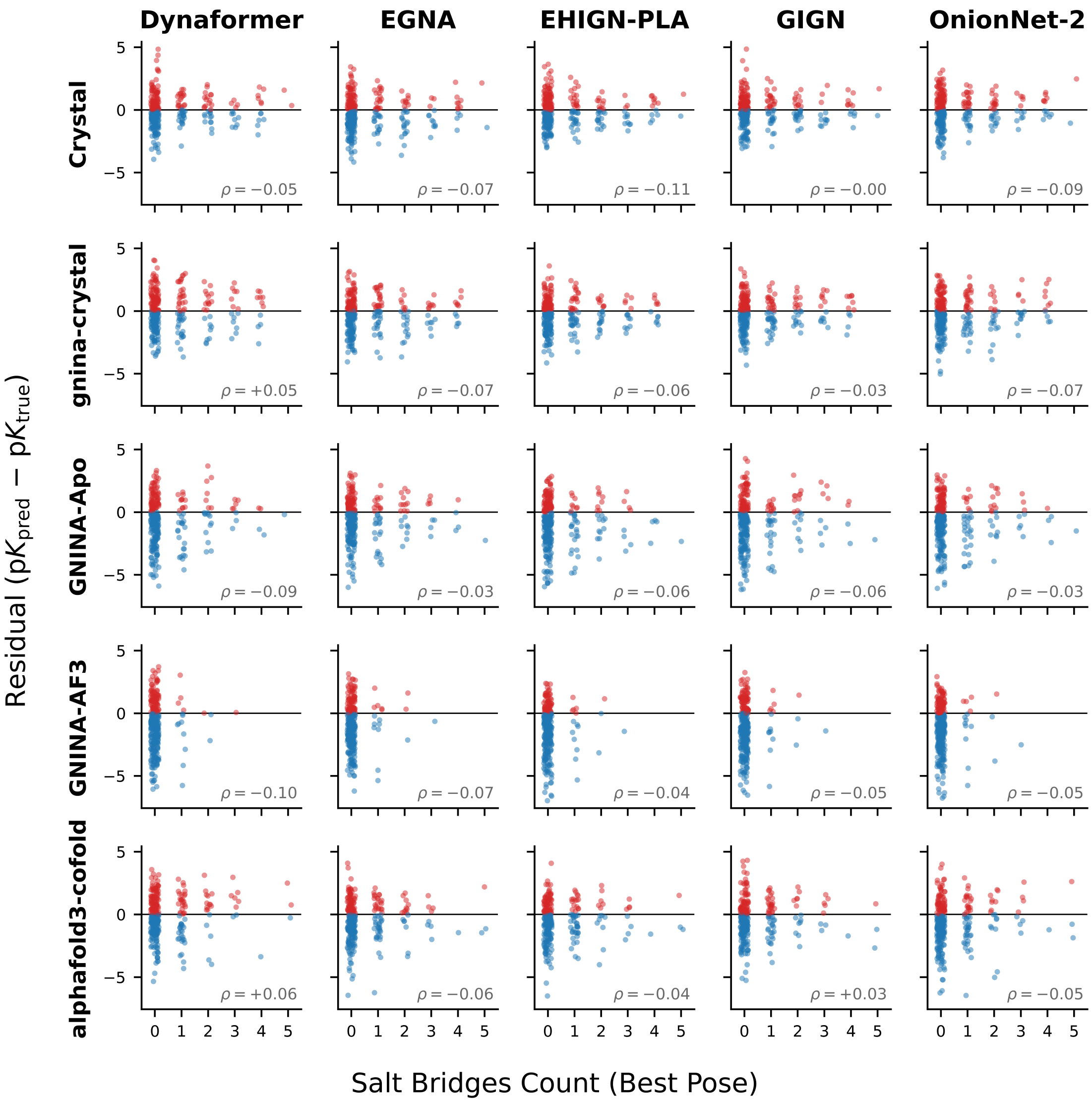

Figure S13: Per-complex prediction residual vs. salt bridges (PLIP, best pose) across all structure sources (rows) and PLBAP models (columns). Spearman ρ between count and absolute residual is annotated. No significant associations were observed across any model–source combination.

Halogen Bonds

No significant associations were observed for any model–source combination. Halogen bonds are rare across CASF-2016 and most complexes have none. See Figure S14.

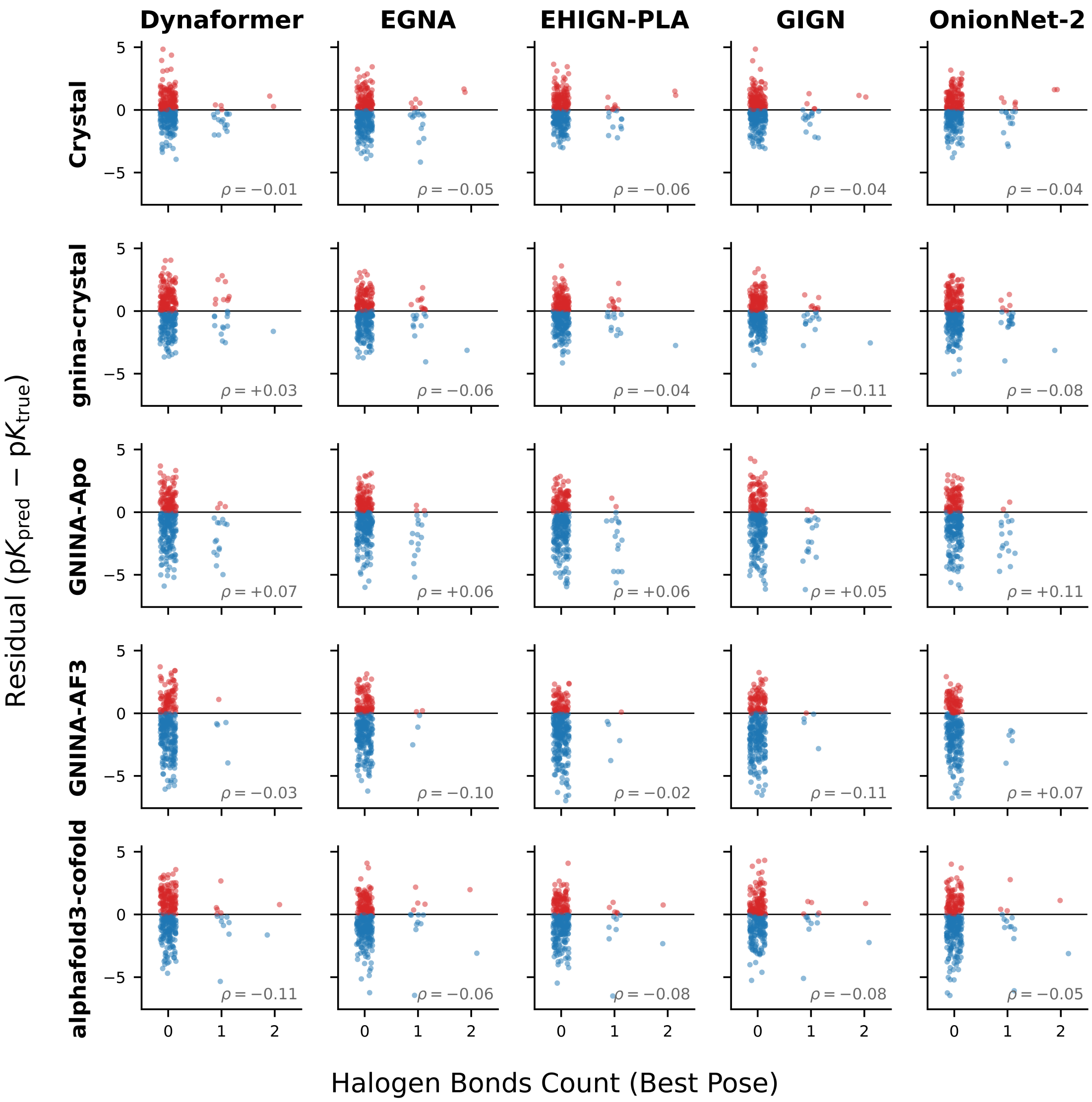

Figure S14: Per-complex prediction residual vs. halogen bonds (PLIP, best pose) across all structure sources (rows) and PLBAP models (columns). Spearman ρ between count and absolute residual is annotated. No significant associations were observed across any model–source combination.

References

[1] Lin, S.; Huang, B.; Zhao, L.-l.; Xu, F.; Pan, D.; Chen, X.; Lin, S. A Python program to merge Sanger sequences: an update. *PeerJ* **2024**, *12*, e18363.

[2] Michaud-Agrawal, N.; Denning, E. J.; Woolf, T. B.; Beckstein, O. MDAnalysis: a toolkit for the analysis of molecular dynamics simulations. *Journal of computational chemistry* **2011**, *32*, 2319–2327.

[3] Abramson, J.; Adler, J.; Dunger, J.; Evans, R.; Green, T.; Pritzel, A.; Ronneberger, O.; Willmore, L.; Ballard, A. J.; Bambrick, J.; others Accurate structure prediction of biomolecular interactions with AlphaFold 3. *Nature* **2024**, *630*, 493–500.

[4] Su, M.; Yang, Q.; Du, Y.; Feng, G.; Liu, Z.; Li, Y.; Wang, R. Comparative assessment of scoring functions: the CASF-2016 update. *Journal of chemical information and modeling* **2018**, *59*, 895–913.

[5] Wang, R.; Fang, X.; Lu, Y.; Yang, C.-Y.; Wang, S. The PDBbind database: methodologies and updates. *Journal of medicinal chemistry* **2005**, *48*, 4111–4119.

[6] Feng, Z.; Chen, L.; Maddula, H.; Akcan, O.; Oughtred, R.; Berman, H. M.; Westbrook, J. Ligand Depot: a data warehouse for ligands bound to macromolecules. *Bioinformatics* **2004**, *20*, 2153–2155.

[7] Passaro, S.; Corso, G.; Wohlwend, J.; Reveiz, M.; Thaler, S.; Somnath, V. R.; Getz, N.; Portnoi, T.; Roy, J.; Stark, H.; others Boltz-2: Towards accurate and efficient binding affinity prediction. *BioRxiv* **2025**,

[8] McNutt, A. T.; Li, Y.; Meli, R.; Aggarwal, R.; Koes, D. R. GNINA 1.3: the next increment in molecular docking with deep learning. *Journal of Cheminformatics* **2025**, *17*, 28.

[9] O'Boyle, N. M.; Banck, M.; James, C. A.; Morley, C.; Vandermeersch, T.; Hutchison, G. R. Open Babel: An open chemical toolbox. *Journal of cheminformatics* **2011**, *3*, 33.

[10] Adasme, M. F.; Linnemann, K. L.; Bolz, S. N.; Kaiser, F.; Salentin, S.; Haupt, V. J.; Schroeder, M. PLIP 2021: Expanding the scope of the protein-ligand interaction profiler to DNA and RNA. *Nucleic Acids Research* **2021**, *49*, W530–W534.

[11] Yasumitsu, Y.; Ohue, M. Generation of appropriate protein structures for virtual screening using AlphaFold3 predicted protein–ligand complexes. *Computational and Structural Biotechnology Reports* **2025**, 100057.
